# PI3K-AKT activation resculpts integrin signaling to drive filamentous tau-induced proinflammatory astrogliosis

**DOI:** 10.1186/s13578-023-01128-x

**Published:** 2023-09-27

**Authors:** Peng Wang, D. Eric Anderson, Yihong Ye

**Affiliations:** 1 Laboratory of Molecular Biology, National Institute of Diabetes, Digestive, and Kidney Diseases, National Institutes of Health, Bethesda, MD 20892 USA; 2 Advanced Mass Spectrometry Core, National Institute of Diabetes, Digestive, and Kidney Diseases, National Institutes of Health, Bethesda, MD 20892 USA

**Keywords:** tau, Integrin, αV/β1, PI3 kinase/PI3K, AKT, Focal adhesion kinase/FAK, Astrogliosis, Alzheimer’s disease/AD, Tauopathy, Neuroinflammation

## Abstract

**Background:**

Microtubule-binding protein tau is a misfolding-prone protein associated with tauopathies. As tau undergoes cell-to-cell transmission, extracellular tau aggregates convert astrocytes into a pro-inflammatory state via integrin activation, causing them to release unknown neurotoxic factors.

**Results:**

Here, we combine transcriptomics with isotope labeling-based quantitative mass spectrometry analysis of mouse primary astrocyte secretome to establish PI3K-AKT as a critical differentiator between pathogenic and physiological integrin activation; simultaneous activation of PI3K-AKT and focal adhesion kinase (FAK) in tau fibril-treated astrocytes changes the output of integrin signaling, causing pro-inflammatory gene upregulation, trans-Golgi network restructuring, and altered secretory flow. Furthermore, NCAM1, as a proximal signaling component in tau-stimulated integrin and PI3K-AKT activation, facilitates the secretion of complement C3 as a main neurotoxic factor. Significantly, tau fibrils-associated astrogliosis and C3 secretion can be mitigated by FAK or PI3K inhibitors.

**Conclusions:**

These findings reveal an unexpected function for PI3K-AKT in tauopathy-associated reactive astrogliosis, which may be a promising target for anti-inflammation-based Alzheimer’s therapy.

**Supplementary Information:**

The online version contains supplementary material available at 10.1186/s13578-023-01128-x.

## Background

Astrocytes, the most abundant glial cells in the central nervous system (CNS), perform diverse functions: Key roles relevant to CNS homeostasis include glutamate and ion balance, neurotransmitter recycling, cholesterol metabolism, and the blood-brain barrier maintenance. Moreover, as a major immune responsive cell type in the CNS, astrocytes can either promote or restrain inflammation in response to environmental cues, which explains their broad implications in different neurological disorders such as Alzheimer’s disease (AD), Parkinson’s disease (PD) etc. [1, 2]. Under pathological conditions, the communication between astrocytes and other CNS-resident cells (neurons, microglia, and oligodendrocytes) or CNS-infiltrating cells (peripheral leukocytes) could be a crucial driver of disease progression [3, 4].

In response to injuries, toxic insults, or pathogen infection, astrocytes undergo extensive changes in morphology, gene expression, and function. This process is generally referred to as “astrogliosis” [5]. Although the mechanism of astrogliosis is not fully understood, reactive astrocytes have now been appreciated as a critical regulator of disease-associated CNS inflammation [6]. Recent advances in single-cell RNA sequencing, single-nucleus RNA sequencing, and spatially resolved transcriptomics have established high-resolution reactivity maps for astrocytes in healthy and disease conditions, revealing global transcriptome remodeling and posttranslational modulation associated with the expression of various cell surface molecules and secretory immune modulators [7–10]. Emerging evidence suggests astrogliosis, as a heterogeneous process, can be influenced by both intrinsic factors and the brain microenvironment in such that subtle variations in signaling input and the cell status may result in either a beneficial anti-inflammation or a detrimental pro-inflammation outcome. While environmental insults are known to induce disease-associated astrogliosis, little is known about the intrinsic factors that dictate astrocytic reactivity [5].

A common pathological hallmark of neurodegenerative diseases is the accumulation of misfolded proteins, which can oligomerize to form various inclusions in the CNS. Moreover, many disease-associated neurotoxic proteins undergo neuron-to-neuron transmission in a prion-like manner by cycles of release and uptake [11]. Once released into the cell exterior, disease-causing proteins such as α-synuclein, tau, and Aβ could also encounter non-neuronal cells like astrocytes given their sheer volume in the CNS. Indeed, several Aβ species and α-synuclein preformed fibrils (PFFs) are known to activate disparate receptors to induce the pro-inflammatory NFκB signaling in astrocytes [12–14]. Along this line, we previously reported that filamentous human tau activates integrin αV/β1 in primary mouse astrocytes, converting them to a neurotoxic state with the expression of a cohort of pro-inflammatory A1 genes upregulated [15, 16]. The implication of integrin in tau-associated astrogliosis is further supported by a translation-associated RNAseq analysis of brains from AD and tauopathy mice, which identified integrin binding and TNFα activation as molecular signatures of astrogliosis in these animals [17].

Integrins mediate cell-cell and cell-extracellular matrix (ECM) interactions during animal development [18]. The ligation of integrins to their ligands generally promotes cell attachment to the ECM, which activates a broad array of signaling activities in cellular proliferation, cytoskeletal reorganization, and other pro-survival processes [19]. In this regard, it is intriguing why tau-activated integrin in astrocytes causes a pro-inflammatory pathogenic outcome.

By profiling the transcriptome and secretome of filamentous tau-treated mouse primary astrocytes (PAs), we show that integrin activation, when coupled to PI3K-AKT activation, yields a completely different functional output in PAs compared to integrin activation alone; Astrocytes are now converted to a reactive state with altered secretory system and increased release of complement C3 and other pro-inflammatory molecules such as cytokines and chemokines. This process is facilitated by NCAM1, a cell surface facilitator of integrin signaling. Importantly, inhibiting either PI3K or FAK is sufficient to mitigate tau fibril-induced astrogliosis, establishing them as potential targets for tauopathy treatment.

## Results

### Tau-induced integrin signaling is distinct from physiological integrin activation

**Fig. 1:**
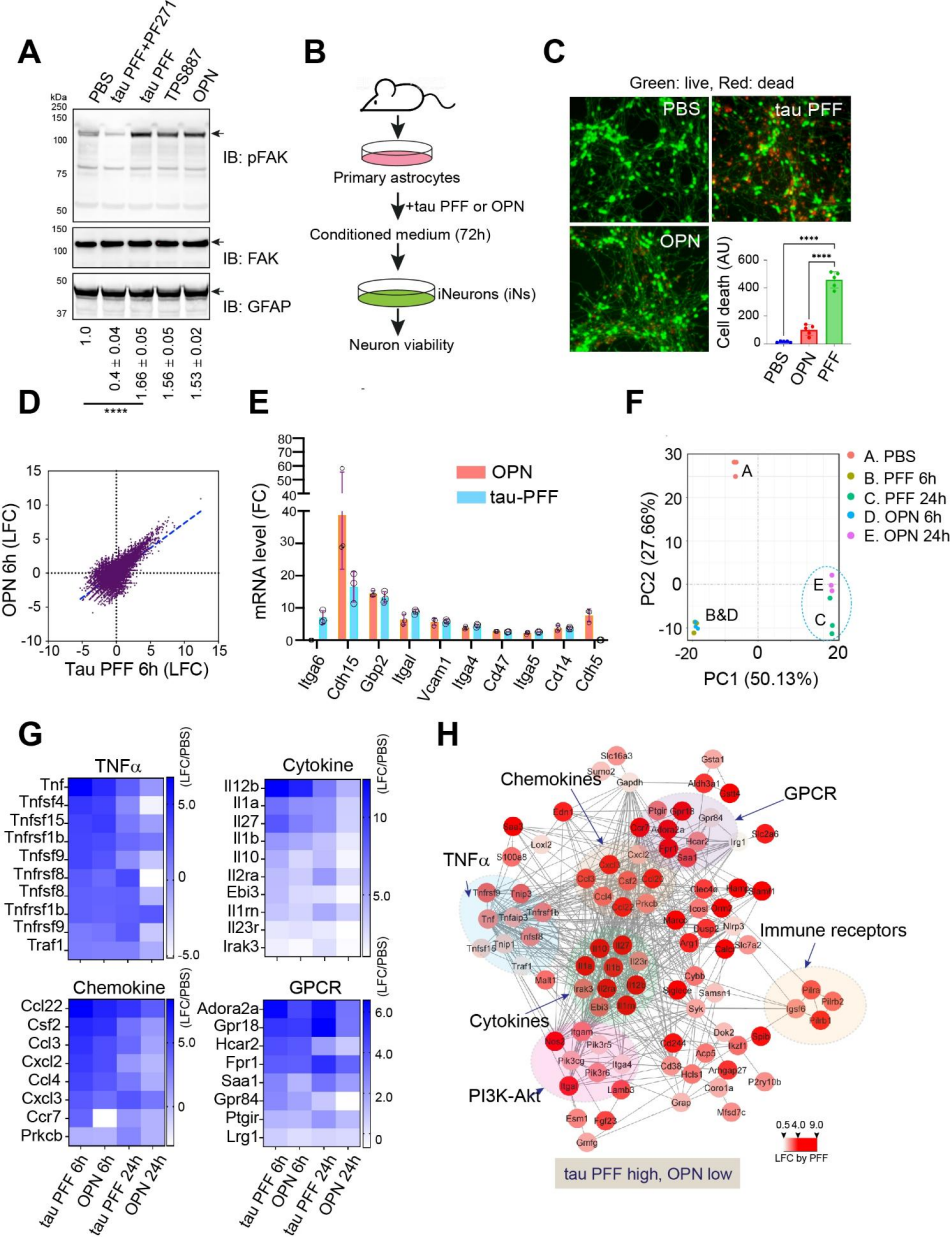
Tau PFF-induced integrin signaling is distinct from physiological integrin activation. (**A**) Immunoblotting (IB) shows the phosphorylation of FAK at Tyr 397 (pFAK) in primary astrocytes (PAs) treated as indicated for 2 h. Total FAK and astrocyte marker GFAP were used as loading controls. PF271, PF-562271; TPS887, brain extract from a 12-month-old tau-P301S transgenic mouse. The numbers indicate the relative level of pFAK ± SD (n = 3 biological repeats) as determined by densitometry. ****, p < 0.0001 by One-way ANOVA. (**B**) Schematic diagram of the cell toxicity assay (**C**) iPSC-differentiated iNeurons treated with the indicated condition medium (CM) from PAs treated with PBS, tau PFF or OPN (see methods) were stained with a green-fluorescent dye to label viable cells and a red-fluorescent dye to label dead cells. The graph shows the quantification of cell death as indicated by red fluorescent dots. Error bars indicate means ± SD. ****, p < 0.0001 by one-way ANOVA with Dunnett’s multiple comparison test. n = 3 biological repeats. AU, arbitrary unit. (**D**) Tau PFF and OPN induce similar gene expression changes. LFC, Log (2) Fold Change. (**E**) OPN (20 μg/ml) and tau PFF (200 nM) activate integrin signaling. qRT-PCR test of selected integrin target genes in tau PFF- and OPN-treated astrocytes (6 h). Error bars indicate means ± SD. n = 3 biological repeats. (**F**) Principal component analysis (PCA) of tau PFF- and OPN-induced gene expression changes. (**G**) Heat maps showing gene expression changes (LFC normalized by PBS-treated conditions) for the indicated pathways. The heat map represents the average of 3 biological repeats. (**H**) A functional interaction map of genes activated more by tau PFF than OPN. The color indicates the relative LFC by tau PFF compared to PBS-treated samples. The scale indicates fold change in log2 (LFC)

To distinguish integrin signaling triggered by preformed tau fibrils (PFF) from that activated physiologically, we first measured phosphorylated Focal Adhesion Kinase (FAK) in mouse PAs treated with either tau PFF or a physiological integrin ligand, Osteopontin (OPN). We chose OPN because previous studies have established it as a physiological ligand capable of engaging multiple integrin receptors including αV/β1 [20], which was recently identified as a primary receptor for tau PFF in PAs [15]. FAK is a plasma membrane-bound kinase [18], whose autophosphorylation at tyrosine 397 in the kinase domain recruits other downstream kinases and adaptors to transmit integrin signaling [21]. Immunoblotting showed that both OPN and tau PFF induced FAK phosphorylation at Tyr397 (Fig. 1A). Likewise, filamentous tau purified from aged tau transgenic mouse brains (TPS887) also activated p-FAK (lane 4). As anticipated, tau-induced FAK phosphorylation was ablated when cells were co-treated with a FAK specific inhibitor PF-562271 (PF271) (lane 2 vs. 3). Together, these results confirm integrin activation by recombinant tau PFF and tauopathy- derived aggregates. Because of the low yield of brain-derived tau fibrils, we used recombinant tau PFF for the rest of the study.

Since tau PFF-activated PAs release a neurotoxic factor(s) in an αV/β1 dependent manner [15], we tested whether OPN could do so. To this end, we treated PAs with tau PFF, OPN, or phosphate buffer saline (PBS) as a negative control for 6 h. Cells washed extensively were replated in fresh medium for 48 h. Conditioned medium (CM) was then used to treat iPSC-derived neurons (iNs) for 72 h [22]. We then measured cell viability with a fluorescence-based cell viability assay (Fig. 1B). As shown with mouse primary neurons [15], iNs exposed to CM from tau PFF-treated PAs underwent massive cell death (Fig. 1C). By contrast, CM from OPN- and PBS-treated PAs was largely non-toxic. Thus, although tau PFF and OPN both activate FAK, only tau PFF but not OPN converts PAs to a neurotoxic state.

To identify the molecular differentiator(s) of the two types of integrin signaling, we used RNAseq to compare the gene expression profile of tau PFF-treated PAs to that of OPN-treated ones using PBS-treated PAs as a reference. RNAseq analysis identified 1172 up- and 542 down-regulated genes by 6-h PFF treatment (Table S1, fold change larger than 2.0 and adjusted p-value smaller than 0.05). For OPN-treated PAs, 1224 genes were upregulated, whereas 449 were downregulated (Table S2). Strikingly, genes regulated by tau PFF overlap significantly with those affected by OPN, both in direction and fold change (Fig. 1D). Using qRT-PCR, we further confirmed the similar activation of known integrin target genes (Itga6, Cdh15, Gbp2, Itgal, Vcam1, Itga4, Cd47, Itga5, Cd14, Cdh5) by tau PFF and OPN (Fig. 1E). These results suggest that tau PFF and OPN impact PAs via the same integrin receptor.

To understand the differential action of tau PFF and OPN on PAs, we analyzed the transcriptome in PAs exposed to these ligands for 24 h. We found a collection of genes that are more significantly upregulated in tau PFF-treated PAs than in OPN-treated ones (Fig. 1F, Table S3). These genes were also differentially regulated in PAs treated with these ligands for 6 h, although the difference was less apparent than in cells treated for 24 h (Fig. 1G). GO analysis showed that these genes belong to several pro-inflammatory pathways, including the TNFα pathway (Tnf, Tnfsf1b, Tnfsf4, Tnfsf8, Tnfsf9, Tnfsf15, Tnfrsf1b, Tnfrsf8, Tnfrsf9, and Traf1), cytokine (Il12b, Il1a, Il27, Il1b, Il10, Il2ra, Ebi3, Il1rn, Il23r, and Irak3) and chemokine (Ccl22, Csf2, Ccl3, Cxcl2, Ccl4, Cxcl3, Ccr7and Prkcb) signaling, or G protein-coupled receptor signaling (Adora2a, Gpr18, Hcar2, Fpr1, Saa1, Gpr84, Ptgir, and Lrg1) (Fig. 1H). Together, these results suggest that tau PFF but not OPN induces a strong and prolonged inflammatory response in PAs.

### PI3K activation defines a pathogenic element in filamentous tau-induced integrin signaling

**Fig. 2:**
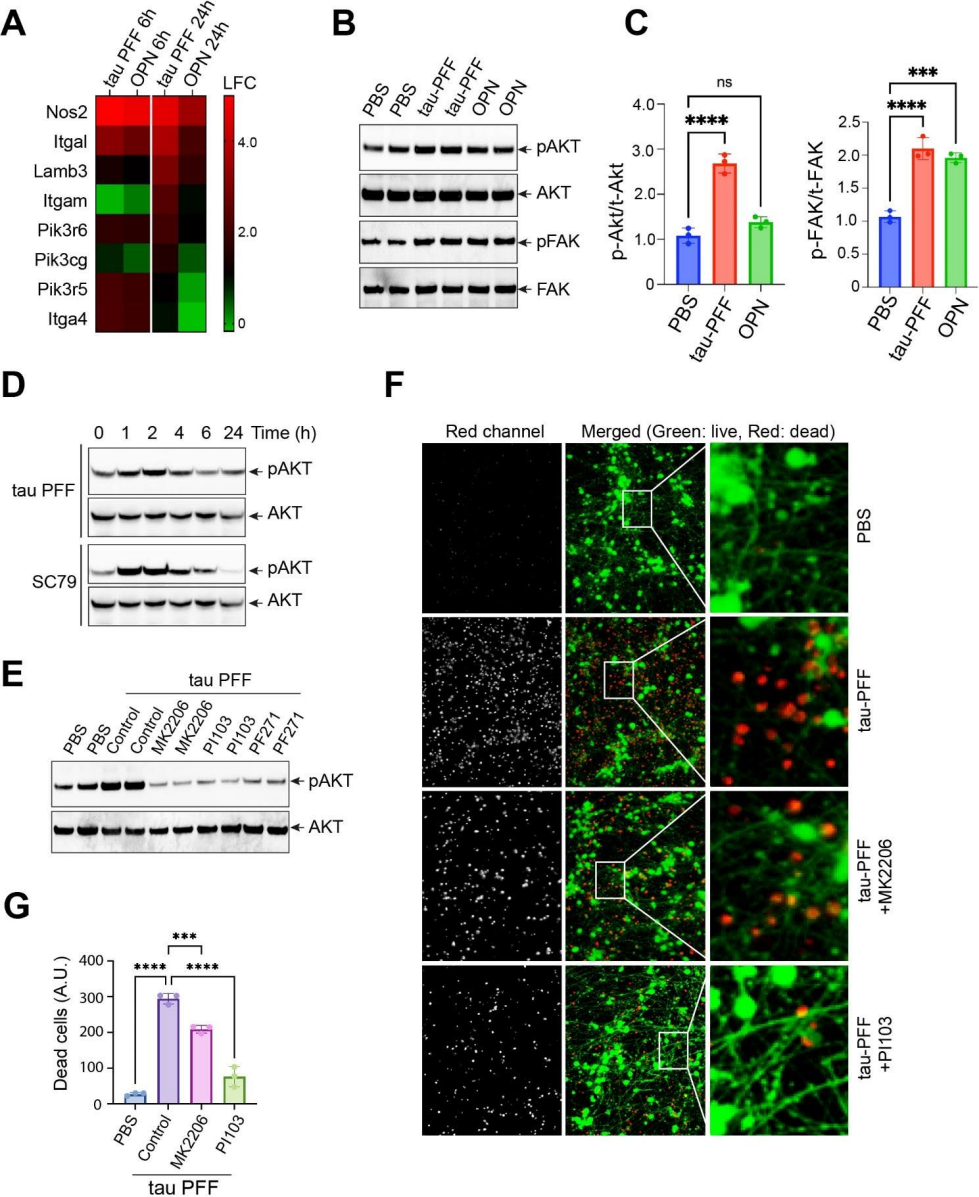
PI3K activation defines a pathogenic element in tau PFF-induced integrin signaling network. (**A**) A heat map shows the induction in LFC for PI3K signature genes in tau PFF- and OPN-treated primary astrocytes normalized to PBS-treated cells. n = 3 biological repeats. (**B**) Immunoblotting analysis of phosphorylation (p) of FAK and AKT in primary astrocytes treated with tau PFF or OPN for 2 h. Total (t) FAK and AKT were used as loading controls. Shown are representative blots from three biological repeats. (**C**) Quantification (mean ± SD) of pAKT and pFAK relative to total tAKT and tFAK (B). ****, p < 0.0001, ****, p < 0.001, ns, not significant by one-way ANOVA. n = 3 biological repeats. (**D**) Representative gels show time chase of AKT phosphorylation in primary astrocytes treated with tau PFF or SC79 (10 μM). (**E**) The effect of the indicated inhibitors on AKT phosphorylation in primary astrocytes treated with PBS or tau PFF. The numbers indicate the ratio of pAKT to tAKT. The data represents three biological repeats. (**F**) iNeurons treated with condition medium from primary astrocytes treated as indicated were stained with a green-fluorescent dye to label viable cells and a red-fluorescent dye to label dead cells. The right panels show enlarged view of the box-indicated areas. (**G**) Quantification of (F). Error bars indicate mean ± SD. ***, p < 0.001; ****, p < 0.0001 by one-way ANOVA. n = 3 biological repeats

Our RNAseq analysis revealed that several genes in the PI3K signaling pathway, including a few PI3K regulatory subunits, are upregulated more significantly by tau PFF than OPN after 24-hour treatment (Fig. 2A), suggesting that PI3K may be more robustly activated in tau PFF-treated PAs. This hypothesis was indeed confirmed by immunoblotting showing that the phosphorylation of AKT (p-AKT Ser_473_), a kinase downstream of PI3K, was significantly upregulated by tau PFF but not by OPN. In contrast, FAK phosphorylation was activated similarly by tau PFF and OPN (Fig. 2B, C). A time course study showed that the maximum p-AKT, detected ~ 2 h after tau PFF treatment, was comparable to that in cells treated with SC79, a brain-penetrable AKT activator (Fig. 2D). After 2 h, the level of p-AKT reduced over time in both tau PFF- and SC79-treated cells. As anticipated, treating PAs with MK2206, an allosteric AKT inhibitor, or PI103, a multi-targeted PI3K inhibitor, significantly blunt PFF-induced p-AKT (Fig. 2E). Additionally, the FAK inhibitor PF-562271 also abolished p-AKT induction by tau PFF. These results suggest an integrin-FAK-PI3K-AKT signaling axis that is more active in tau PFF-treated PAs than in OPN-treated ones.

We next performed the iNeuron toxicity assay to test whether the activation of PI3K-AKT contributes to tau fibril-induced astrogliosis. We previously showed that the co-treating PAs with tau PFF and the FAK inhibitor PF-562271 significantly attenuates the neurotoxicity of the CM [15]. Likewise, CM harvested from PAs pre-treated with MK2206 or PI103 was significantly less toxic to iNs than the CM from PAs treated with tau PFF alone (Fig. 2F, G). Thus, the activation of PI3K-AKT appears to reshape the integrin signaling network, converting a benign cellular signaling to a pathogenic one.

### PI3K-AKT activation drives a pro-inflammatory integrin signaling in PAs

**Fig. 3:**
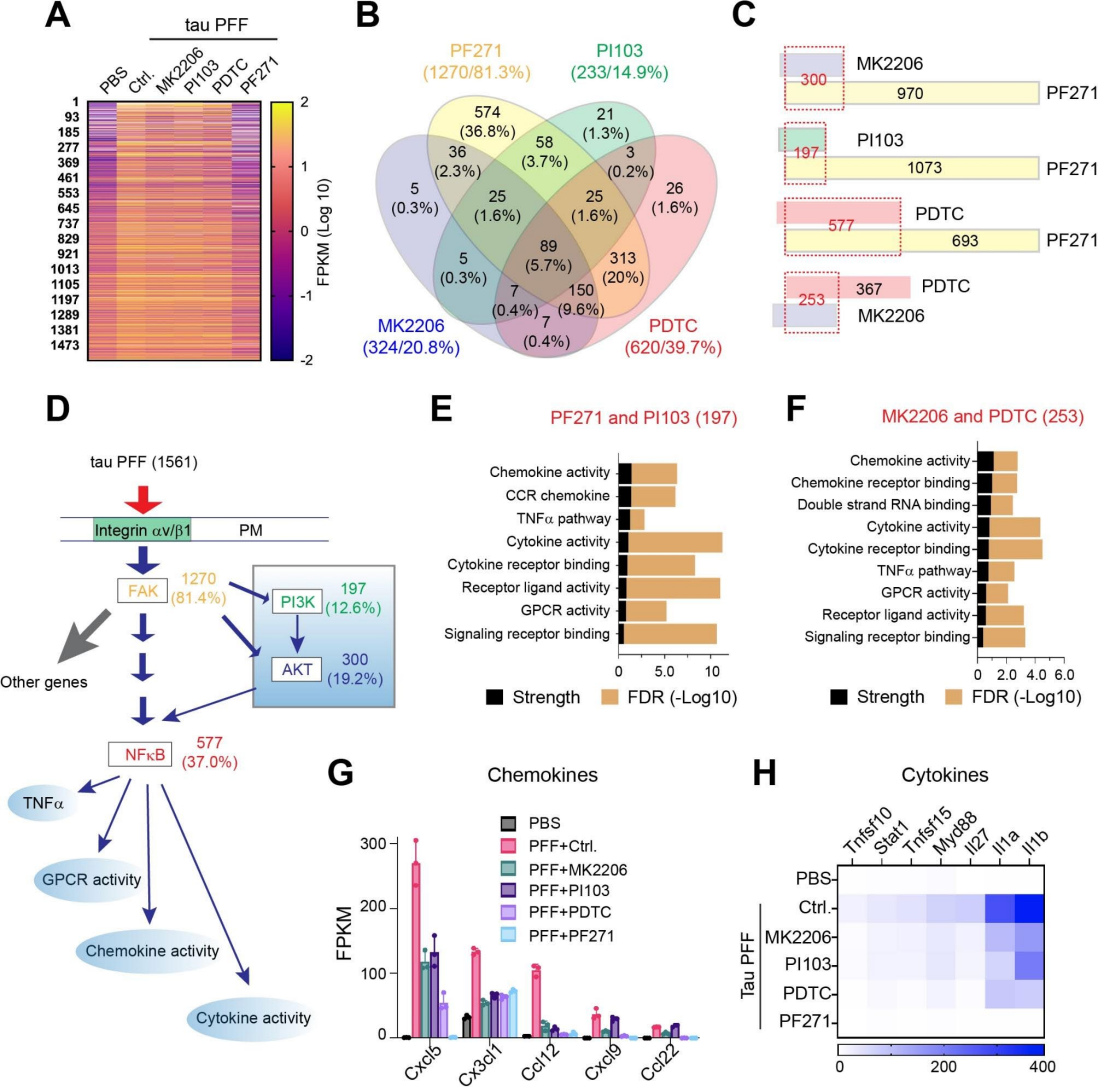
PI3K-AKT activation drives a pro-inflammatory integrin signaling output in primary astrocytes. (**A**) A heat map showing the 1561 genes induced by tau PFF in primary astrocytes and their expression in control PBS-treated cells or in tau PFF treated cells that were exposed to the indicated inhibitors. n = 3 biological repeats. (**B**) A Venn diagram showing the relative impact of the indicated inhibitors on tau PFF-induced gene expression. The numbers indicate the number/percentage of genes whose induction was reverted by the inhibitor. (**C**) A paired comparison showing the relative impact of the inhibitors on tau PFF-induced gene expression. The numbers indicate the number of genes whose induction was reverted by the inhibitor. (**D**) A working model showing the integrin-FAK-PI3K-AKT-NFκB signaling axis that determines a pro-inflammatory response in tau PFF-treated astrocytes. (**E, F**) STRING biological process analysis of tau PFF-induced genes commonly regulated by FAK and PI3K (**E**) or those by both AKT and NFκB (**F**). FDR, false discovery rate. (**G**) The expression of selected chemokine family members in astrocytes treated with tau PFF together with the indicated inhibitors. Error bars indicate means ± SD. n = 3 biological repeats. (**H**) A heat map shows the relative expression of cytokines in primary astrocytes treated with tau PFF and the indicated inhibitors

To determine how PI3K-AKT signaling re-sculpts the integrin pathway in tau PFF-treated PAs, we performed another RNAseq experiment in PAs treated with tau PFF together with PF-562271, PI103, or MK2206 (Table S4). We also included an NFκB inhibitor (PDTC) since our previous study suggested that NFκB acts downstream of FAK to mediate tau PFF-induced astrogliosis [15]. Consistent with integrin being the master regulator in tau PFF-treated PAs, the FAK inhibitor PF-562271 almost completely blunted the effect of tau PFF on gene expression, reverting the expression of 1270 upregulated genes (81.3%) (Fig. 3A). By contrast, the PI3K, AKT, or NFκB inhibitor only reverted the expression of 14.9%, 20.8% and 39.7% genes, respectively, in tau PFF-treated PAs (Fig. 3B). As expected, most genes reverted by MK2206 (300 out of 324), PI103 (197 out of 233), or PDTC (577 out of 620) are also reverted by PF-562271 (Fig. 3C), suggesting that PI3K, AKT, and NFκB act exclusively downstream of FAK in tau PFF-treated PAs (Fig. 3D). By contrast, while 253 genes (out of 324) reverted by MK2206 are also reverted by PDTC, a significant fraction of the PDTC-affected genes (367 out of 620) are not reverted by MK2206 (Fig. 3C). These results suggest that both AKT-dependent and independent factors contribute to NFκB-regulated genes downstream of FAK (Fig. 3D). GO analysis of the genes commonly regulated by FAK and PI3K (Fig. 3E) or those affected by both AKT and NFκB (Fig. 3F) highlighted the molecular signature of TNFα-dependent inflammation, including many chemokine- and cytokine-encoding genes (Fig. 3G, H). Collectively, our transcriptome analyses suggest that activation of the PI3K-AKT-NFκB signaling axis in the context of FAK activation is a crucial molecular determinant for a pro-inflammatory signaling output in tau PFF-treated PAs.

### Filamentous tau stimulates conventional and unconventional protein secretion in PAs

**Fig. 4:**
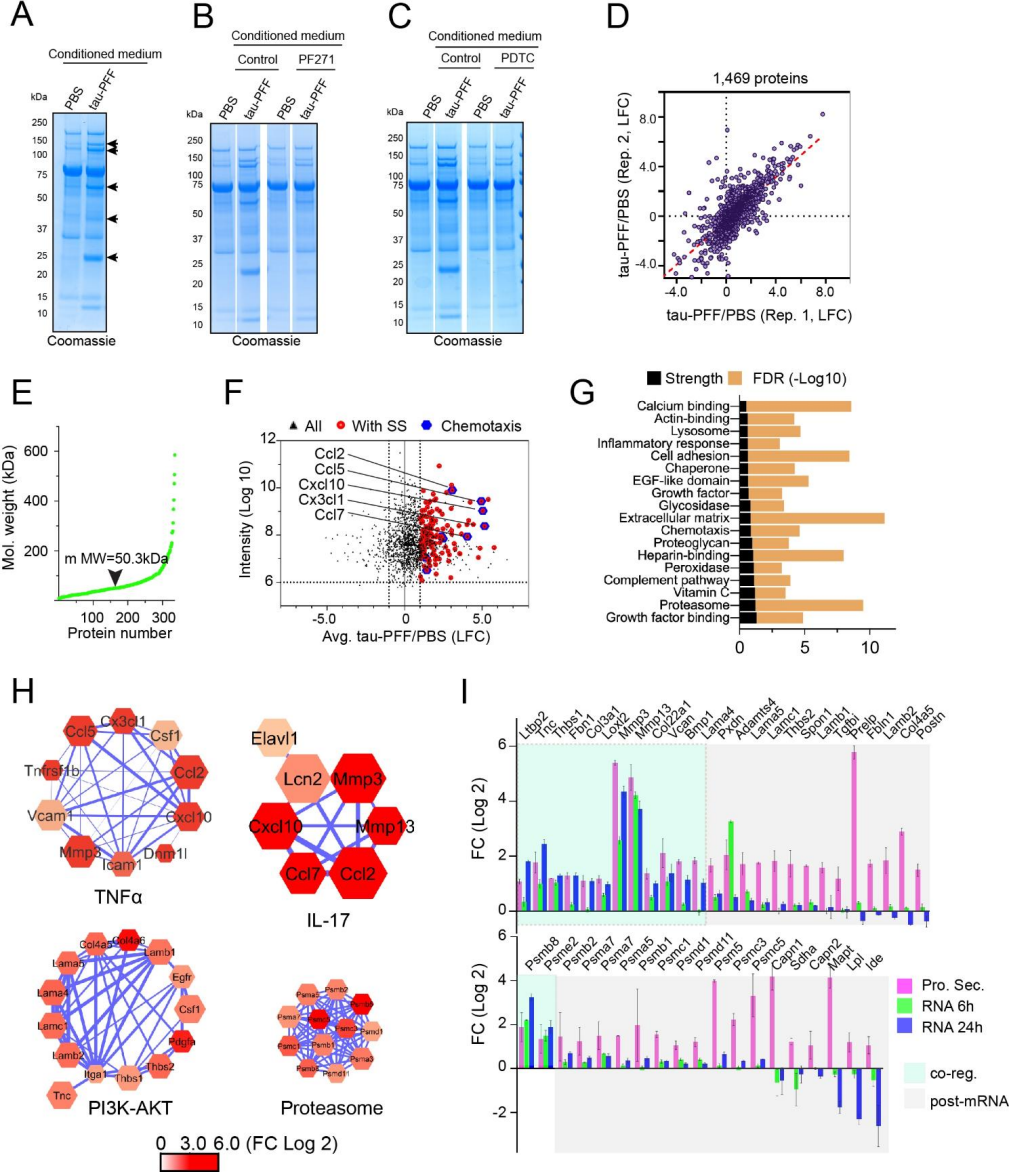
Filamentous tau stimulates conventional and unconventional protein secretion in PAs. (**A-C**) Representative Coomassie blue-stained gels show increased protein secretion in PAs treated with tau PFF (A), which was significantly inhibited in cells pretreated with the FAK inhibitor PF-562271 (PF271) (B), or the NFκb inhibitor PDTC (C). The arrows in (A) indicate significant protein bands only detected in tau PFF-treated condition medium. For each condition, conditioned medium (1 mL) was concentrated 10 times using Amicon Ultra 3 K centrifugal filters, and 20 μL concentrated medium was loaded. Shown are representative gels from three biological repeats. (**D**) Two independent mass spectrometry analyses of conditioned medium from tau PFF-treated PAs compared to PBS-treated ones (Rep., Replication). (**E**) The molecular weight distribution of proteins whose secretion was induced by at least 2-fold by tau PFF in PAs. (**F**) A scatter plot showing the Log Fold Change (x-axis) and the relative intensity (abundance) of tau PFF-induced secretory proteins. Red dots represent proteins bearing a signal sequence (SS). Blue dots highlight a few induced chemokines. (**G**) STRING network analysis of tau PFF-induced secretory proteins. FDR, false discovery rate. (**H**) Examples of the STRING networks of proteins of the indicated pathways. The node size indicates the relative abundance in CM from tau-PFF treated PAs. The color indicates LFC caused by tau PFF. (**I**) Tau PFF-induced protein secretion is partially regulated by a post-transcriptional mechanism. The green shaded genes (co-reg., co-regulated) show similar induction in protein secretion (Pro. Sec.) and mRNA expression

Since CM from tau PFF-treated PAs is toxic to neurons, we wished to determine how tau PFF modulates protein secretion from PAs. To this end, PAs were treated with tau-PFF or PBS for 6 h. After an extensive wash to remove unbound tau fibrils, cells were incubated in a protein-free medium. CM was then harvested, concentrated, and analyzed by SDS-PAGE. Coomassie blue staining revealed more secreted proteins in tau PFF-treated CM than in control CM (Fig. 4A). When PAs were pretreated with the FAK inhibitor PF-562271 or the NFκB inhibitor PDTC, tau PFF-induced protein secretion was largely abolished (Fig. 4B, C). Tau PFF treatment in immunopanning-purified PAs also induced global protein secretion, which was similarly inhibited by PF-562271, PDTC or by *Talin1* knockdown (figs. S1 A-C). Together, these results indicate that tau PFF alters protein secretion via integrin and NFκb activation.

To identify the neurotoxic factor(s) released by tau PFF-treated PAs, we used isotope labeling-based quantitative mass spectrometry to compare the secretome of control- and tau PFF-treated PAs [23]. To maximize protein coverage, we first separated concentrated CM by HPLC into 15 fractions. Each fraction was subject to trypsin digestion and differential isotope labeling. The resulting peptides were identified by mass spectrometry. Analyses of two independent biological repeats identified 1469 proteins reproducibly presented in the CM (Fig. 4D). Among them, 336 proteins with a median molecular weight of 50 kDa was elevated in CM of tau PFF-treated PAs by at least 2-fold (Fig. 4E, F, Table S5). A significant fraction of the enriched proteins (39.3%) carries an amino-terminal signal sequence, which should be secreted through the canonical endoplasmic reticulum (ER)-Golgi pathway (Fig. 4F). However, ~ 60% of the identified proteins do not have a signal sequence. They are likely released by unconventional protein secretion because most of these proteins are not abundant cytosolic proteins (e.g., HSP70, HSP90, VCP etc.), which would be the predominant species if protein release was caused by cell death. Consistent with the elevated inflammatory signatures by our transcriptome analyses, many chemokines of the C-C motif chemokine ligand (CCL) family were upregulated in CM from tau PFF-treated PAs (Fig. 4F). These factors would attract immune cells from the peripheral circulation if present in brains. GO pathway analysis further confirmed that proteins involved in the TNFα pathway and inflammatory response are enriched in the CM from tau PFF-treated PAs (Fig. 4G, H). Additionally, proteins of the IL17 pathway (e.g. MMP3, MMP13, CXCL10), the complement system, and extracellular matrix proteins (ECM) are also among the most significantly upregulated pathways in the CM from PFF-treated PAs (Fig. 4H, fig. S1D), in accord with the increasing appreciation of these factors in AD development [24] (see discussion). Moreover, several proteins encoded by PI3K-AKT target genes are also enriched in the CM from PFF-treated PAs (Fig. 4I), further corroborating our transcriptome analysis. Surprisingly, mass spectrometry also identified a large number of proteasome subunits in the medium in response to tau PFF treatment (Fig. 4H). Immunoblotting with available proteasome antibodies confirmed the presence of the  α7 subunit in the CM (fig. S1E). Comparing fold change (FC) of tau PFF-treated secretome to that of mRNA expression showed that only ~ 40% of the secreted proteins are associated with a similar level of mRNA induction, suggesting that tau PFF-stimulated protein secretion results from a combinatory regulation of gene transcription and a post-transcriptional mechanism (see below). Altogether, our analyses suggest that integrin ligation by tau PFF alters both gene transcriptional machinery and the secretory systems in PAs, upregulating protein secretion via both conventional and unconventional protein secretion pathways.

### The complement C3 released by tau PFF-treated PAs is a neurotoxic factor

**Fig. 5:**
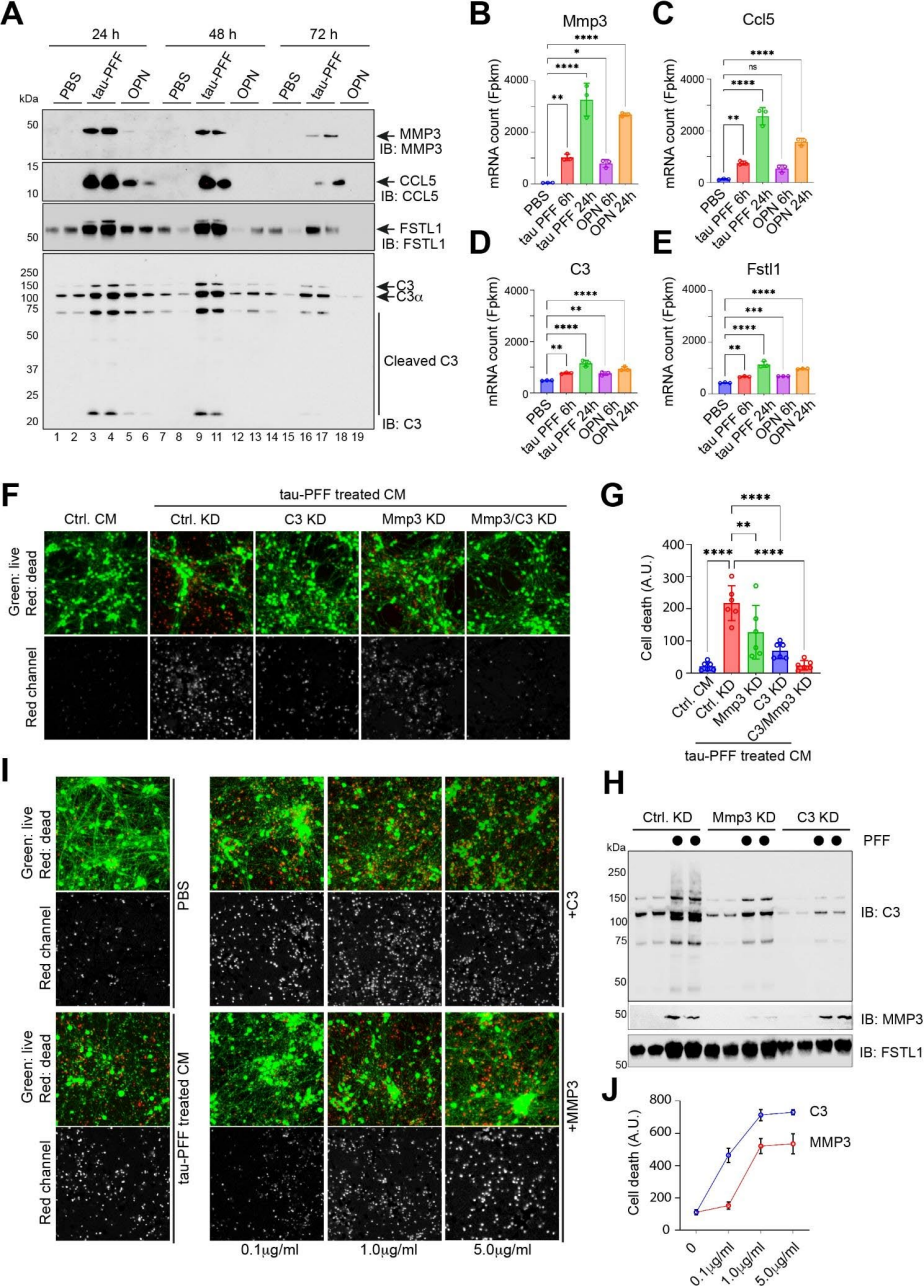
Complement C3 released by tau PFF-treated PAs is a primary neurotoxic factor. (**A**) Immunoblotting analysis of the indicated secretory proteins in conditioned medium from primary astrocytes exposed to tau-PFF, OPN, or PBS. The blots represent three biological repeats. (**B-E**) The mRNA expression of the indicated genes from the RNAseq experiment in Fig. 1. Error bars indicate means ± SD. *, p < 0.05; **, p < 0.01, ****, p < 0.0001 by one-way ANOVA. n = 3 biological repeats. (**F, G**) iNeuron viability test after treatment with condition medium (CM) from tau PFF-treated wild type control (Ctrl.) astrocytes or astrocytes with the indicated gene knockdown (KD). The graph in (G) show the quantification of cell death. Error bars indicate means ± SD. *, **, p < 0.01, ****, p < 0.0001 by one-way ANOVA. n = 3 biological repeats. (**H**) Immunoblotting analysis of the indicated proteins in the condition medium from wild type primary astrocytes or C3- or MPP3-knockdown astrocytes treated with tau PFF. Shown is a representative gel from 3 biological repeats. (**I**) Neurotoxicity testing after iNeurons were treated with recombinant protein MMP3 or C3 at the indicated concentrations. Cells treated with PBS or tau PFF in parallel were used as negative and positive controls, respectively. (**J**) Quantification of cell death in (I). Error bars indicate means ± SD, n = 3 biological repeats

We used immunoblotting to validate a few selected proteins identified by mass spectrometry. Among the top hits, we focused on complement C3 (C3), matrix metalloprotease 3 (MMP3), C-C motif chemokine ligand 5 (CCL5), and Follistatin-like 1 (FSTL1) because these proteins have been implicated in AD pathology by previous studies [25–28], and also because well-characterized antibodies are commercially available. We measured their abundance in CM harvested at different time points following 6-h exposure to tau-PFF, OPN, or PBS. Consistent with the mass spectrometry results, tau PFF treatment induced a robust secretion of all four proteins, whereas OPN had no effect on MMP3 secretion and only weakly induced the secretion of CCL5, FSTL1, and C3 (Fig. 5A). Additionally, the secretion of these factors persisted longer in tau-treated cells than in OPN-treated cells. Importantly, several proteolytically cleaved bioactive C3 fragments were more enriched in the CM from PFF-treated PAs than those from OPN- or PBS-treated cells, indicating tau PFF induces high complement activities. RNAseq analysis showed that the mRNA of Mmp3 and Ccl5 were highly upregulated by tau PFF and OPN whereas C3 and Fstl1 mRNAs were only modestly induced by tau PFF (Fig. 5B-E), suggesting that the secretion of these proteins is regulated via both transcriptional and post-transcriptional mechanisms. RNAseq analysis further revealed that several additional genes of the complement system such as C2, Cfb, C1ra, C1S1, C3ar1 were also induced by tau PFF, whereas other genes of the complement system including C1ql1, C1qc, C1qa, C1qb, C5ar1, Cfh were downregulated (Table S6). Thus, tau PFF appears to activate a selection of complement genes.

Since C3 and MMP3 were suggested as glia-released factors non-autonomously impacting neuronal viability [25, 26], we tested whether they are responsible for the neurotoxic activity in tau PFF-treated CM. We took two approaches. First, we knocked down C3- and MMP3-encoding mRNAs either individually or in combination for 48 h using lentiviral particles expressing shRNAs targeting these genes. PAs were then treated with PBS or tau PFF for 6 h and replated in tau-free medium for 48 h. We then used CM from these cells to treat iNs. The CM from tau-treated C3 knockdown PAs was significantly less toxic than that from similarly treated control PAs (Fig. 5F, G). Knockdown of Mmp3 in PAs also reduced the neurotoxicity of CM after tau PFF treatment, but the effect was weaker than C3 knockdown. Combined knockdown of C3 and Mmp3 completely eliminated the CM toxicity compared to individual knockdown, suggesting an interplay between these two factors. Consistent with this notion, we unexpectedly found by immunoblotting and qRT-PCR that Mmp3 knockdown reduced the expression and secretion of both Mmp3 and C3, whereas C3 knockdown only mitigated C3 expression and secretion (Fig. 5H, fig. S2). These results suggest C3 as a major toxic factor in tau PFF-treated PA CM, but also reveal an unexpected functional link between MMP3 and C3.

To further confirm the neurotoxicity of C3 and MMP3, we treated iNs with recombinant C3 or MMP3 proteins at different concentrations. Cell viability analysis showed that C3 effectively induced cell death in iNs even at low concentrations (Fig. 5I, J). By contrast, MMP3 only showed toxicity at high doses. These results demonstrate C3 as a major neurotoxic factor in the CM from tau PFF-treated PAs.

### Tau PFF alters the trans-Golgi network in an integrin-dependent manner

**Fig. 6:**
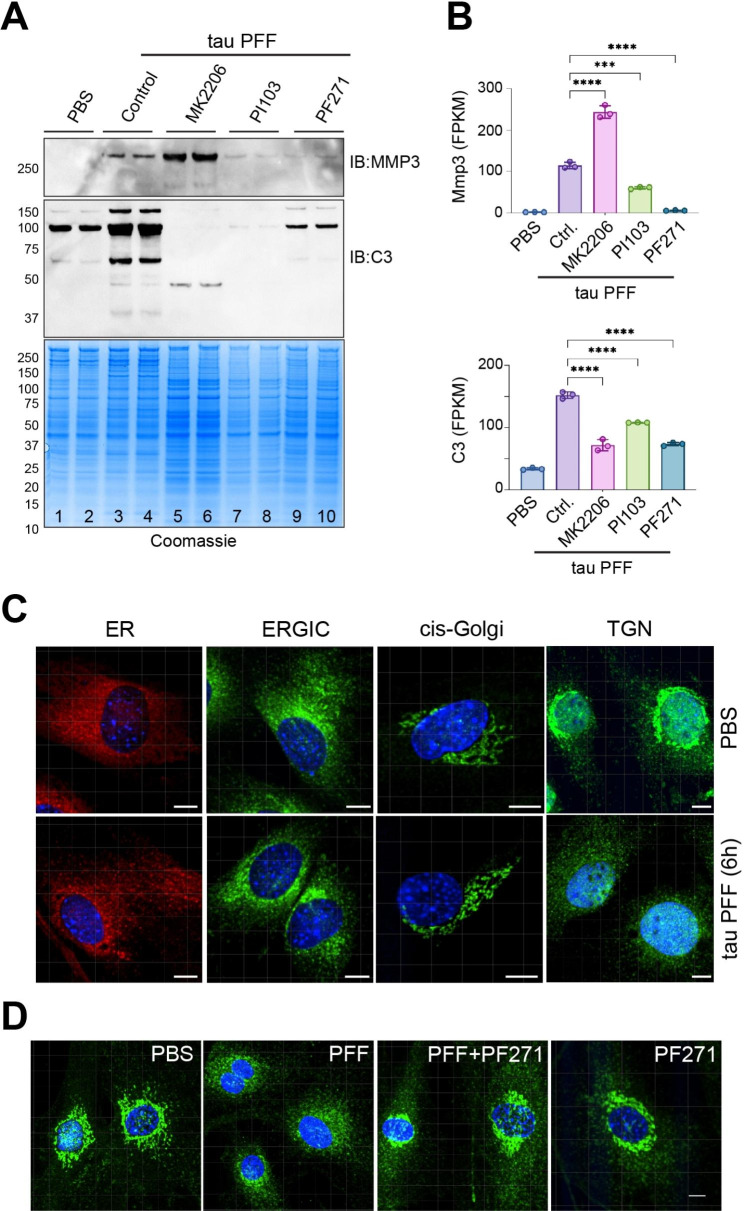
Tau PFF alters the trans-Golgi network in an integrin-dependent manner. (**A**) Representative immunoblotting and Coomassie staining of protein secretion in primary astrocytes exposed to tau-PFF together with the indicated inhibitors. n = 3 biological repeats. (**B**) The mRNA expression of the indicated genes from the RNAseq study in Fig. 3. Error bars, means ± SD. ***, p < 0.001, ****, p < 0.0001 by one-way ANOVA. n = 3 biological repeats. (**C**) Tau PFF treatment alters the trans-Golgi network (TGN) in PAs. Primary astrocytes treated with either PBS or tau PFF for 6 h were stained with antibodies against the following organelle markers, Calreticulin (ER), ERGIC53 (ERGIC), GM130 (cis-Golgi), VTI1B (TGN) together with a DNA dye (blue). Scale bard, 10 μm. (**D**) Tau PFF-induced Golgi fragmentation is inhibited by PF562271 (PF271). Cells treated with PBS, tau PFF alone or in combination with PF271 or PF271 alone were stained by VTI1B antibodies (green) and DAPI (blue). Scale bar, 10 μm. Images represent 3 biological repeats

We next test whether the release of C3 and MMP3 from tau PFF-treated PAs is controlled by the FAK-PI3K-AKT signaling axis using inhibitors targeting these molecules. For C3, pre-treating cells with either PF-562271, PI103, or MK2206 (targeting FAK, PI3K, and AKT, respectively) significantly reduced its secretion (Fig. 6A, lanes 5–10 vs. lanes 3, 4). By contrast, for MMP3, while PF-562271 and PI103 reduced its secretion, the AKT inhibitor MK2206 increased the secretion (Fig. 6A). Because the level of C3 and MMP3 secretion is generally correlated with their mRNA levels under these conditions (Fig. 6B), the change in C3 and MMP3 secretion is probably regulated mostly at the transcriptional level. Interestingly, co-treating cells with OPN and SC79 was not sufficient to induce C3 and MMP3 secretion (fig. S3A), suggesting other signaling components in addition to PI3K-AKT signaling pathway are required to activate protein secretion in tau PFF-treated PAs.

Given that the secretion of many proteins is induced by tau PFF independent of their mRNA expression (Fig. 4I), we tested whether tau PFF treatment could modulate the secretory system. To this end, we stained tau PFF-treated PAs with antibodies that labeled distinct membrane compartments involved in protein secretion. We did not observe any significant changes in the early secretory compartments such as the ER, the ER-Golgi intermediate compartment (ERGIC), or the cis-Golgi network in tau PFF-treated PAs (Fig. 6C). However, the staining pattern of a trans-Golgi network (TGN) protein VTI1B was dramatically altered: in ~ 60% of control-treated cells, TGN is concentrated in a perinuclear region as a result of Golgi stacking (Fig. 6C). By contrast, most tau PFF-treated PAs (~ 80%) display the TGN as dispersed vesicles throughout the cytoplasm (Fig. 6C), a phenotype reminiscent of that observed in AD animal models [29, 30]. The Golgi fragmentation phenotype could be largely rescued when cells were pre-treated with PF-562271, but not by the PI3K inhibitor PI103 (Fig. 6D, fig. S3B). These results suggest that tau PFF treatment alters the secretory system in an integrin-dependent manner (see discussion).

### NCAM1 assists αV/ β1 in tau PFF-induced astrogliosis

**Fig. 7:**
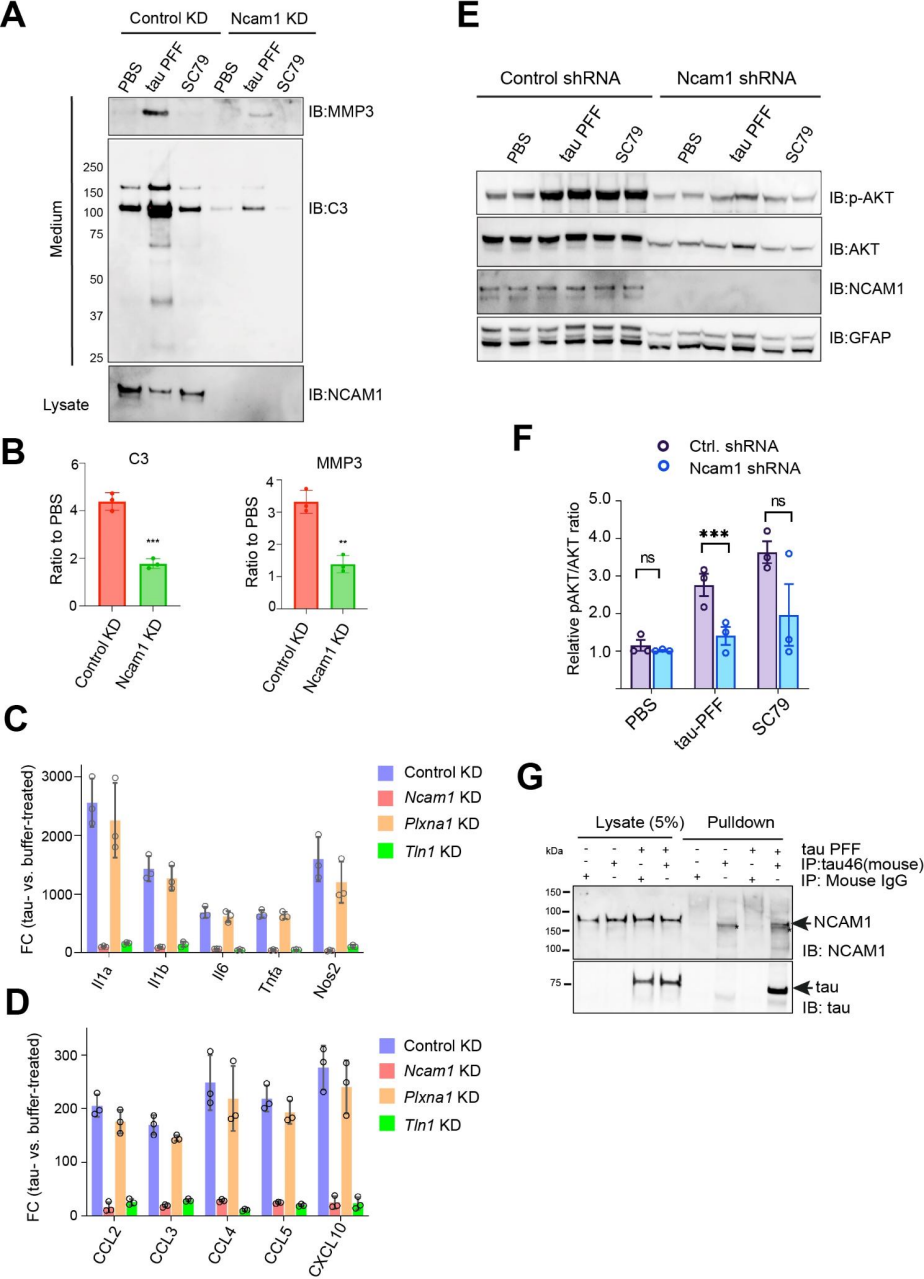
NCAM1 assists αV/ β1 in tau PFF-induced astrogliosis. (**A**) Conditioned medium from wild type or Ncam1 knockdown astrocytes treated with the indicated agents was analyzed by immunoblotting. The lower panel shows the protein level of NCAM1 in the corresponding cell lysates. The gels represent one of the three biological repeats. (**B**) The graphs show the quantification of C3 and MMP3 secretion in tau PFF-treated control and NCAM1 knockdown cells in three independent experiments. **, p < 0.01, ***, p < 0.001 by unpaired Student’s t-test. n = 3 biological repeats. (**C, D**) qRT-PCR analysis of the indicated cytokine (**C**) and chemokine genes (**D**) in wild type PAs or in PAs with the indicated gene knockdown after treatment with tau PFF. The graphs show Log Fold Change normalized to PBS-treated cells. Error bars, means ± SD. (**E, F**) Representative gels show that Tau PFF-induced phosphorylation of AKT (pAKT) requires NCAM1. Wild type PAs or Ncam1 knockdown astrocytes were treated with tau PFF or SC79 as a control. Cell lysates were analyzed by immunoblotting (**E**). Note that Ncam1 knockdown reduces total AKT and GFAP possibly because of a growth defect associated with Ncam1 knockdown [55]. pAKT normalized to total AKT was quantified in (**F**). Error bars, means ± SD. ***, p < 0.001 by unpaired student’s t-test. n = 3 biological repeats. (**G**) Co-immunoprecipitation shows a physical interaction between tau PFF and endogenous NCAM1. Astrocytes were treated with tau PFF or PBS as a control. Cell lysates were subjected to immunoprecipitation with either a control IgG or a tau specific antibody (tau-46). Cell lysates and precipitated proteins were analyzed by immunoblotting. Asterisk indicates a non-specific band. Shown are blots representing 3 biological repeats

To identify other signaling components involved tau PFF-induced astrogliosis, we screened additional cell surface molecules labeled by tau-APEX2 in our proximity-based ligation study. In addition to integrin αV/b1, three other cell surface molecules, including PLXNA1, CD44, and neural cell adhesion molecule 1 (NCAM1) were readily labeled by tau-APEX2 [15], suggesting that they are in close proximity to tau PFF when it activates integrin. We used a lentivirus-based knockdown approach to express pre-validated shRNAs targeting these genes individually in PAs and found that only knockdown of Ncam1 could diminish tau PFF-induced secretion in PAs similarly as Talin1 knockdown (Fig. 7A, fig. S4A, B). Immunoblotting further confirmed that knockdown of Ncam1 inhibited the secretion of C3, MMP3, and CCL5 (Fig. 7A, B, fig. S4A). qRT-PCR analysis showed that Ncam1 knockdown also blunted tau-induced upregulation of pro-inflammatory genes, including cytokines and chemokines (Fig. 7 C, D). Importantly, after tau PFF treatment, Ncam1 knockdown cells had reduced p-AKT compared to PFF-treated control cells (Fig. 7E, F), suggesting that NCAM1 is also required for tau-induced AKT activation, pro-inflammatory transcriptome induction, and increased protein secretion. Immunoprecipitation of tau PFF from cell extracts using a tau-specific antibody co-precipitated endogenous NCAM1 (Fig. 7G), demonstrating an interaction between these proteins. These results suggest NCAM1 as a critical signaling component for tau PFF-induced proinflammatory responses in PAs.

## Discussion

### PI3K-AKT activation is a major pro-inflammatory driver in tau-induced integrin signaling

Integrins are a family of 24 heterodimeric transmembrane receptors that, upon ligand binding, assemble into a large molecular complex named the “adhesome”. These complexes contain essential signaling components, such as Talin-1 and FAK, which link integrin activation to cytoskeleton remodeling. Integrin signaling generally promotes cell survival by mediating cell adhesion to various ECM components and cell-cell interactions [31]. However, we recently reported that filamentous human tau employs integrin αV/b1 as a major receptor to convert astrocytes into a neurotoxic proinflammatory state [15]. Why integrin activation in astrocytes by filamentous tau generates a pathogenic signaling output is unclear. Our study suggests that the PI3K-AKT signaling axis is a critical element that resculpts the integrin signaling in favor of pro-inflammatory astrogliosis because AKT phosphorylation at Ser473 is preferentially upregulated by tau PFF but not a physiological integrin ligand, and because AKT inhibition reverses the pro-inflammatory gene expression pattern in tau PFF-treated astrocytes. Intriguingly, previous studies have suggested seemingly contradictory roles for PI3K-AKT in inflammation depending on the cellular context [32–35]. The fact that the same signaling component can be pro-inflammatory in astrogliosis but anti-inflammatory in macrophage activation suggests that context-specific regulators play a critical role fine-tuning the PI3K-AKT signaling upon integrin activation.

Integrin activation in conjunction with a growth stimulus has been linked to PI3K-AKT activation, which promotes attachment-dependent cell survival [36]. Integrin activation alone may activate PI3K during tumor metastasis [37, 38], because upon phosphorylation on Y397, FAK can bind to PI3K [39]. However, since OPN-treated astrocytes activate FAK without significant AKT activation, additional signaling components are required in addition to FAK autophosphorylation to turn on the PI3K-AKT axis in tau PFF-treated PAs. Moreover, while integrin and PI3K-AKT activation are required for pro-inflammatory astrogliosis, activating these signaling components is not sufficient to induce protein secretion, suggesting additional missing components (fig. S4C). In this regard, the large number of molecules in the adhesome may provide many opportunities for FAK to interface with other signaling activities, regulating tau PFF-associated astrogliosis.

### Transcriptome and secretome remodeling in tau PFF-activated astrocytes

Our study shows that tau PFF treatment in astrocytes dramatically alters the gene expression landscape in an integrin-dependent manner. FAK mediates most transcriptional changes, but only a subset of genes require the entire FAK-PI3K-AKT-NFκB signaling axis, including many cytokine- and chemokine-encoding genes key to inflammation regulation. Thus, the downstream NFκB activation appears to be essential for tau-induced reactive astrogliosis, a finding consistent with widely documented pro-inflammatory role of NFκB [40]. Tau PFF-induced astrogliosis also causes the fragmentation of the TGN, a membrane compartment functioning at the crossroad between conventional and unconventional protein secretion pathways [41]. Golgi fragmentation is known to accelerate vesicle budding from the Golgi, and thus increase the secretory flow [42]. Indeed, we found that tau-activated astrocytes secret more proteins. In AD animal models, Golgi fragmentation in response to Aβ accumulation is mediated via phosphorylation of the Golgi stacking regulator GRAPS65 by CDK5 [30], a kinase that can be activated by integrin signaling [43]. These reports are consistent with our finding that TGN vesiculation in response to tau PFF requires FAK activation. Collectively, these results establish integrin as a master regulator in cellular response to external proteotoxic stress.

Our unbiased transcriptome and secretome analyses have identified many proteins, including both conventional and unconventional secretory cargos, whose expression and secretion are upregulated by tau PFF. Among them, the CCL family chemokines are the most significant ones. Astrocytes are a known source of chemokine release in the CNS, while several chemokine receptors were identified on microglia and monocytes. Recent studies indicate chemokines as critical messengers regulating the communications between astrocytes and other immune cells within the neural vesicular unit (NVU) [3, 44]. Our findings suggest that the remodeling of astrocytic secretome by tau PFF may disrupt the NVU homeostasis, causing functional changes in neurons and the blood–brain barrier to worsen tauopathy.

Moreover, we show that tau PFF treatment activates the secretion of complement C3 from astrocytes, which is a critical driver of neurodegeneration. The mRNA expression of C3 and its receptor C3aR are upregulated in AD patients with tauopathy, and C3aR ablation attenuates tau pathology and reverses neuroinflammation in a tau-transgenic mouse AD model [25]. Likewise, inactivation of C3 rescued plaque-associated pathology in APP mice and ameliorates neuron loss in Tau-P301S mice [45]. These findings underscore a direct communication between astrocytes and neurons, mediated by C3 and its receptor, which contributes to AD- and tauopathy-associated neurodegeneration.

MMP3 is another factor whose expression and secretion are highly induced by tau PFF, and therefore, may represent another disease-associated change in NVU. Consistent with this notion, a recent report showed that MMP3 secretion is highly induced in microglia co-cultured with tau aggregate–containing neurons or in brains from patients with tauopathy [46]. Elevated CSF MMP3 was also detected in individuals with increased risk for AD, together with increased tau and phosphor-tau [47], and Mmp3 expression was shown to correlate with progressive neurodegeneration. Additionally, in AD brain, Mmp3 expression is high in astrocytes around neurofibrillary tangles and amyloid plaques [48]. Although our study does not support a direct role for MMP3 in neurodegeneration, astrocyte-released MMP3, as a protease capable of re-sculpting the ECM, may modulate neuroinflammation, and thus, prime neurons into a state more sensitive to C3-induced cell death.

### NCAM1 facilitates tau-induced astrogliosis

Why does tau PFF but not OPN activate PI3K-AKT? A key difference between tau PFF and a physiological integrin ligand is that the latter usually functions as a monomer or dimer whereas tau fibrils are large oligomers. It is possible that tau fibril may ligate multiple integrin receptors, driving them into super clusters, which lead to a different signaling output. Integrin super-clustering can indeed form under conditions of inside-out signaling, which is thought to potentiate signaling intensity [49]. Alternatively, oligomerize tau may engage additional cell surface molecules in addition to integrin αV/β1. Consistent with this model, we find that tau PFF also binds to NCAM1, which is required for tau-induced protein secretion and pro-inflammatory gene activation. NCAM1, also called CD56, is a membrane-bound glycoprotein expressed on the surface of neurons and glia. Upregulation of NCAM1 was reported after neural injury [50], which inhibits astrocyte proliferation [51]. NCAM1, if recruited to the adhesome upon tau PFF engagement, may change how adhesome networks with other signaling axes, allowing FAK to activate PI3K.

## Conclusions

Our studies reveal the activation of PI3K-AKT signaling downstream of tau fibril-induced integrin activation in mouse primary astrocytes. Combined activation of integrin with PI3-AKT appears essential for tau-induced reactive astrogliosis, which alters the gene expression profile, the Golgi morphology, and the secretome composition in astrocytes. These findings suggest an astrogliosis-associated pro-inflammatory signaling axis that may be targeted to restrict neuroinflammation in AD with tauopathy.

## Methods

### Animals and reagents

Wild-type C57BL/6J and transgenic tau-P301S mice (PS19) [52] expressing human tau-P301S driven by the mouse *prion* promoter (The Jackson Lab: Stock# 008169; Strain Name: B6;C3-Tg(Prnp-MAPT*P301S)PS19Vle/J) were obtained from The Jackson Lab. Tau-P301S mice were aged to develop a neurodegenerative phenotype as previously reported at ~ 10–11 months [52]. All animals were maintained in accordance with the animal care standards of the National Institutes of Health. All reagents are listed in table S7. Animal studies were conducted following the animal study protocol ASP K117-LMB-17 approved by the NIDDK Animal Care and Use committee.

### Conventional primary astrocyte culture

Primary astrocyte cultures were prepared from cerebral cortices of P2-P5 C57BL/6J wild type mice. Cortices were dissected, stripped of meninges, and digested with 0.25% trypsin at 37 °C in Hank’s Balanced Salt Solution (HBSS) (Thermo Fisher) for 10 min. Trypsinization was stopped by the addition of astrocyte culture medium (DMEM/ F12 50/50, Thermo Fisher) containing 25 mM glucose, 4 mM glutamine, 1 mM sodium pyruvate, and 10% FBS). Single-cell suspension of the digested tissue was obtained by repeated pipetting. Cells were seeded into a 75 ml flask at a density of ~ 4 × 10^5^ cells/cm2 and cultured in astrocyte culture medium at 37 °C in a humidified 5% CO2 incubator. Monolayers of glial cells were obtained 7–10 days after plating. To remove microglia, cultures were gently shaken, and the floating cells (microglia) were removed, resulting in more than 95% pure astrocytes. The remaining astrocytes were incubated for 72 h before lentiviral infection for gene knockdown or drug treatment. Before experiments, astrocytes were dissociated by trypsinization and then reseeded at 4 × 10^5^ cells cm^2^ or 1.5 × 10^5^ cells per well in 24-well or 1.5 × 10^6^ cells per well in 6-well or 2 × 10^5^ cells/cm2 in Labtek imaging dish in DMEM F12 50/50 containing 10% FBS and 1% penicillin-streptomycin. At the onset of each experiment, the medium was changed to regular DMEM without serum and antibiotics.

### Immunopanning-based astrocyte purification

Where indicated, astrocytes were purified by immunopanning from P2-P5 C57BL/6J mice and cultured as previously described [53]. Briefly, cortices were digested by trypsin at 37 °C and then mechanically dissociated to generate a single-cell suspension, which was incubated in successive negative immunopanning plates to remove macrophage and microglia cells before positive selection for astrocytes by an ITGβ5 antibody-coated panning plate. Isolated astrocytes were cultured in a defined, serum-free base medium containing 50% neurobasal, 50% DMEM, 100 U/ml penicillin, 100 μg/ml streptomycin, 1 mM sodium pyruvate, 292 μg/ml L-glutamine, 1 x G5 supplement, and the astrocyte-required survival factor HBEGF at 5 ng/ml.

### Differentiation and growth of iNeurons

6 well plate or 10 cm dishes coated with poly-D-lysine were used for differentiation. To this end, plates were placed it in 37 °C incubator for 30 min to 1 h or overnight in a coating solution prior to beginning dissociation or re-plating. Cells were dissociated from plates with 1 ml Accutase for 6 well plate or 4 ml for a 10 cm dish (8–10 min/37°C) and gentle pipetting. Cells were then resuspended in 1 ml of N2 medium (Knockout DMEM/F12, N2 supplement, non-essential amino acids, Gluta-MAX, Chroman I). 6–8 × 10^6^ iPSC cells were seeded per 10-cm dish in 10–12 ml of N2 media. Repeated medium exchange with N2 + doxycycline medium for three days. On day 4, pre-differentiated neurons were ready to be re-plated. Before coating, freshly prepared poly-L-ornithine (PLO) was used at 0.1 mg/ml to coat plates for at least 1 h or overnight at 37 °C. After cells were dissociated by Accutase, cells were collected by centrifugation and then resuspended in Cortical Neuron Culture Medium (CNCM) (BrainPhys neuronal medium, B27 + supplement, GDNF, BDNF, NT-3, laminin, doxycycline) and counted. 1.5 million cells were plated onto each well in a PLO-coated 6 well plate or 6 million for 10 cm dish with 12 mL CNCM. Medium was changed twice per week.

### Purification of tau protein

Recombinant human (tau 2N4R isoform) containing 2 N-terminal inserts and four microtubule binding repeats was expressed using the pET29b vector in the *E.coli* strain BL21. From a culture volume of 10-liter, cell pellet was resuspended in ice cold cell resuspension buffer (20 mM MES, 1 mM EGTA, 0.2 mM MgCl2, 5 mM DTT, 1 mM PMSF). Cells were disrupted by sonication, and NaCl was added to a final concentration of 500 mM. Cell suspension was boiled for 20 min to denature most proteins including lipopolysaccharide (LPS) but not tau. Denatured proteins and insoluble cell debris were sedimented by centrifugation at 127,000 × g for 40 min at 4 °C. The supernatant was dialyzed overnight twice in the cation exchange chromatography buffer A (20 mM MES, 50 mM NaCl, 1 mM EGTA, 1 mM MgCl2 with 2 mM DTT, 0.1 mM PMSF at 4 °C under constant stirring. The cleared sample after centrifugation at 127,000 × g for 40 min at 4 °C was loaded onto a cation-exchange chromatography column (mono-Q). The bound tau protein was eluted with a linear gradient of 0–60% of the final concentration of buffer B (20 mM MES, 1 M NaCl, 1 mM EGTA, 1 mM MgCl2, 2 mM DTT, 0.1 mM PMSF) over six column volumes. Fractions containing tau were pooled and concentrated by an ultrafiltration device (Millipore 10 kDa MW cutoff) to a final volume of 1 ml. Concentrated tau protein was further fractionated by a size exclusion column in phosphate buffer saline (PBS) with 1 mM DTT, flash-frozen in liquid nitrogen and stored at -80 °C.

### Preparation of tau helical filaments and tauopathy-derived aggregates

For tau polymerization is, 50 μM recombinant tau was incubated in PBS containing 12.5 μM heparin, a protease inhibitor cocktail (10 μg/ml leupeptin, 5 μg/ml chymostatin, 3 μg/ml elastatinal, and 1 μg/ml pepstatin), and 2 mM DTT at 37 °C for two weeks. Heparin induces tau polymerization. Fresh DTT (1 mM) was added to the solution every 24 h to maintain the reducing condition. After polymerization, tau filaments were dialyzed extensively with PBS to remove heparin and DTT. Negative stain and transmission electron microscopy were used to confirm filament assembly [15].

To purified tau aggregates from tauopathy mice, 8–12 g of cortex was homogenized by a Dounce homogenizer in nine volumes (v/w) of a high-salt buffer (10 mM Tris-HCl, pH 7.4, 0.8 M NaCl, 1 mM EDTA, and 2 mM dithiothreitol (DTT), with a protease inhibitor cocktail, phosphatase inhibitor phoStop, and PMSF) containing 0.1% sarkosyl and 10% sucrose. The brain homogenates were centrifuged at 10,000 g for 10 min at 4 °C. Pellets were re-extracted twice using the same buffer, and the supernatants from all three extractions were filtered and pooled. Additional sarkosyl was added to the pooled supernatant to 1%. After 1-h shaking at room temperature, samples were centrifuged again at 300,000 g for 60 min at 4 °C. The resulted 1% sarkosyl-insoluble pellets, which contain pathological tau proteins, were washed twice in PBS and then resuspended in PBS by passing through 27-G needles. The resuspended sarkosyl-insoluble pellets were sonicated briefly (20 pulses at ∼0.5 s/pulse) followed by centrifugation at 100,000 g for 30 min at 4 °C. Most protein contaminants were partitioned into the supernatant, but tau aggregates remaining in the pellet fraction. The pellets were resuspended in PBS at one half of the pre-centrifugation volume, sonicated with 20–60 short pulses (∼0.5 s/pulse), and spun at 10,000 g for 30 min at 4 °C to remove large debris. The final supernatants, which contained tauopathy-derived aggregates were used in the study.

### RNA isolation and gene expression analyses

Cells were treated with tau PFF at 200 nM for 6 or 24 h. This concentration was chosen as it induced FAK phosphorylation similarly as OPN. Whenever indicated, cells were co-treated with the following inhibitors: MK-2206 (5 μM), PI103 (5 μM), PF-562271 (1 μM), PDTC (90 μM). RNA isolation was performed using a RNeasy Mini Kit (Qiagen) according to the manufacturer’s protocol. RNA concentration was measured using Nanodrop-1000. Complementary DNA synthesis was performed using an iScript™ Reverse Transcription Supermix (Bio-Rad) according to the manufacturer’s instructions, with a minimal input of 200 ng total RNA. Quantitative PCR (qPCR) was performed using a CFX96 Touch Real-Time PCR Detection System (Bio-Rad) using cDNA amount equivalent to 1–2 ng total RNA during cDNA synthesis. SsoAdvanced Universal SYBR Green Supermix (Bio-Rad) and a 2 pmol/ml mixture of forward and reverse primers were used for 45 cycles of gene amplification. The primers used for qPCR are listed in Supplementary Table 6. GAPDH mRNA was used as an internal reference. The CFX manager software was used to analyze the qPCR results. To calculate fold changes, we used PBS-treated samples as a reference. RNAseq experiments were conducted by Novogene using the standard custom service platform.

### Lentivirus production and infection

For Lentivirus production, two 15 cm dishes of HEK293FT cells were seeded at 40% confluence. On the next day, 1 h prior to transfection, the medium was replaced with 13 ml pre-warmed Opti-MEM medium (Thermo Fisher). Transfection was performed using Lipofectamine 2000 and the PLUS reagent (Thermo Fisher). For each dish, 6.8 μg pCMV-VSV-G, 10.1 μg psPAX2 (Addgene), 13 μg gene-specific lentiviral shRNA plasmids (see Table S7) and 135 μl of PLUS reagent (Thermo Fisher) were added to 4 ml Opti-MEM as mixture A, which is then mixed with mixture B containing 68 μl lipofectamine 2000 and 4 ml Opti-MEM. The complete mixture was incubated for 20 min at room temperature and then added to cells. After 6 h, the medium was changed to 25 ml D10 medium (DMEM medium with 10% FBS and 1% Bovine Serum Albumin) with antibiotics (penicillin/streptomycin, 10 U/ml) for virus production. After 60 h of incubation, virus-containing medium from two culture dishes were combined and centrifuged at 2000 × g at 4 °C for 10 min to pellet cell debris. The supernatant was filtered through a 0.45 μm low protein-binding membrane (Steriflip HV/PVDF, Millipore). To concentrate lentivirus, the cleared supernatant was ultracentrifuged at 47,000 × g for 2 h at 4 °C using the JA25.50 rotor (Beckman). The virus was resuspended overnight in 180 μl D10 medium at 4 °C. Virus was aliquoted, flash-frozen in liquid nitrogen and stored at − 80 °C. For lentiviral infection, 10 μl concentrated was directly added to astrocytes cultured in 6-well. The medium was changed 24 h after infection, and the knockdown efficiency was evaluated by qRT-PCR or immunoblotting 96 h of post-infection.

### Conditioned medium preparation and isotope labeling-based mass spectrometry analysis

Astrocytes were treated with tau PFF 200 nM together with the inhibitors specified in the figure legends. Cells were incubated with a serum free medium containing 0.05% trypsin for 10 min at 25 °C. Cells were washed at least three times to remove the remaining tau. Cells were then incubated with a serum-free and protein-free medium at 37 °C in the incubator for the indicated time periods. After incubation, conditioned medium was collected and concentrated 10 times using Amicon Ultra 3 K centrifugal filters (Millipore) for Western blotting.

For isotope dimethyl labeling-based quantitative mass spectrometry, two independent conditioned medium replicates were processed. To digest the proteins in the conditioned media, EDTA (5 mM), NaDodecanoate (0.5%) and 5 μL of commercial ovalbumin (as a processing control) in 50 mM Tris, 100 mM hydroxy-proline, urea (~ 230 pmol/mL) and DTT (10 mM) were added to each sample. Samples were heated at 60 °C for 15 min. After cooling, three volumes of 0.5 M chloroacetamide was added and the samples were further incubated under foil for 1 h at room temperature. Next, half volume of 1 M β-mercaptoethanol was added to scavenge excess chloroacetamide for 15 min. Then 15 μL of trypsin (4 mg/mL, Promega) was added per milliliter of sample. The samples were mixed thoroughly and incubated at room temperature overnight. Sample processing was done in Waters TruView vials (Waters 186005660CV).

The next morning, samples were acidified at room temperature for 15 min after addition of 1/10th volume of 10% formic acid. These samples were dried down to ~ 500 μL with continuous flow of nitrogen gas at 60 °C. The samples were extracted three times with equal volume of ethyl acetate. After the last extraction, the aqueous phase (bottom) was transferred to a new vial. To maximize peptide yield, 300 μL 0.4% formic acid was added back to the tubes containing ethyl acetate. After mixing and centrifugation, the bottom phase was collected and combined with the first fraction. The vials were placed under nitrogen for 5 min to remove stray ethyl acetate. The vials were then sealed and placed on ice for 10 min.

Each sample was then loaded by centrifugation at 200–300 g at 5 °C onto a flow-matched serial stack of STAGE tips comprising 4x C8 Empore gauge 10 cores resting on top of 4x gauge 10 cores C18 Empore (Tip type Axygen T-350-C-L-R) [54]. The tips were then washed with 150 μL buffer containing 1.6% formic acid and 50 mM ammonium acetate followed by two washes each with 150 μL 1.6% formic acid, and lastly with 150 μL 0.16% formic acid. A small volume of 0.16% formic acid was added to the top of each tip and an aluminum foil “hat” was wrapped around each tip assembly, which was stored overnight at 4 °C.

“Reductive dimethylation” was used to isotopically label amino groups of peptides bound to each STAGE tip as described previously [23]. Briefly, the liquid over the Empore beds was “flicked” out and the order of the tips was reversed (C8 bottom). The tip stack was placed on top of a receiver vial. 50 μL of the respective modification solution (-CH3 for the PBS-treated sample and ^13^CD3 for the tau PFF-treated one. The -CD2H label was used for a condition to be discussed in the future) was applied at 300 g for 5 min [23]. Next, 150 μL of the same modification reagent was added to the respective stack followed by three centrifugations at 100 g for 10 min each and a 300 g spin for 5 min. Next 150 μL of the same modification reagent was added to the respective stack and the tips were spun three times at 100 g for 10 min each followed by a 300 g spin for 5 min. The flow through solution was collected and then acidified by 150 μL of 10% formic acid on ice for 10 min.

The flow through solution was applied to their respective column stack in the original order (C8 top) as described for the initial loading. The columns were washed with three washes of 150 μL 1.6% formic acid, 50 mM ammonium acetate and one wash with 150 μL 1.6% formic acid. The columns were placed back in the centrifuge in reverse order (C18 top) over fresh vials after the centrifuge was preheated to 40 °C. To elute peptides, 150 μL 0.4% formic acid, 40% acetonitrile (heated to 60 °C) was added to the top (C18) column and the solution was forced through the stack by centrifugation at 300 g (up to 15 min). Repeat the elution procedure with a solution of 150 μL 0.4% formic acid, 80% acetonitrile.

After checking the labeling efficiency by running a small amount (~ 2.5%) of the material, equal amount of labeled samples were mixed and loaded onto a pre-equilibrated in-house STAGE tips packed with 8x SCX Empore gauge 10 cores at 200 g. The tips were washed two times with 300 μL 0.4% formic acid 40% acetonitrile and then two washes with 300 μL 0.4% formic acid 80% acetonitrile. The peptides were eluted by applying two 100 μL 5% ammonium hydroxide, 40% acetonitrile.

This eluted samples were heated at 60 °C and dried under a stream of nitrogen gas. 200 μL of 50% acetonitrile was added and the sample was dried again. 25 μL of a 1:1 mixture of the A1 and B1 solutions (see below) was added, and the samples agitated to redissolve the peptides. Then 225 μL of A1 solution was added and the vials were placed in a sonication bath for 5 min. The samples were spun down at 1,000 g briefly before being placed in the auto-sampler of a micro preparation LC system (AKTA-Micro).

A continuous concatenated separation (CONCAT) was internally programmed using the fraction collector operating with a drop delay method. Previous experience had suggested an isotope effect on the pKa of methyl modified amino groups, so when working with “reductive dimethylation”-based samples, we targeted a pH significantly lower than that used for conventional CONCAT separation (~ pH 7.2). HPLC fractionation was performed by mixing two buffers (A1 and B1) containing 10 mM ammonium acetate (from a stock filtered through C18 Empore), 0.018% ammonium hydroxide (diluted from a 28% stock) and 0% (A1) or 80% (B1) acetonitrile. The micro-LC system was configured first with the buffer A1 and the operating flow rate was 100 μL/minute at 50 °C. Fractions were collected into flat bottomed vials (Waters 186005660CV) that had the top centimeter removed by a MicroLux band saw (MicroMark). The vials were thoroughly cleaned to allow faithful delivery of the drops into the vial. The samples were loaded using five 40 μL injections approximately 3 min apart onto a reversed phase column (Waters XBridge BEH 130 C18, 2.1 mm x 15 cm). A multi-section smooth gradient was applied to the column consisting 1-6.4% in 1.47mL, 6.4-10.86% in 2.45mL, 10.86–15.29% in 4.09mL, 15.29–18.71% in 3.03mL, 18.71–22.14% in 2.24mL, 22.14–25.57% in 1.66mL, 25.57-29% in 1.23mL, 29-32.43% in 0.91mL, 32.43–35.86% in 0.67mL, 35.86–39.29% in 0.50mL, 39.29–42.71% in 0.37mL, 42.71–46.14% in 0.27mL, 46.14–49.57% in 0.20mL, 49.57-53% in 0.15mL, and 53–80% in 1.0mL. The flow-through samples were diverted to 15 tubes in five repeated cycles. After fractionation, the samples were transferred to sample vials (Waters 186,005,662), dried down under nitrogen gas, and reconstituted in 50 μL of 0.1% formic acid 2% acetonitrile. Each vial was heated to 50 °C and agitated aggressively, spun down at 1,000 g, and sonicated for 15 min. After another centrifugation, the samples were transferred to the autosampler of a Thermo nLC-1000.

Each of the 15 fractions were then analyzed by LC/MS/MS using the nLC-1000 interfaced with a Thermo Fusion Lumos mass spectrometer with the starting solution being 0.1% formic acid in water. 10 μL of each sample was loaded onto a non-vented 500 cm Easy Spray Column at a pressure of 450 psi. The flow was set to 0.1 μL/minute. After a short washing period, a gradient was performed comprising a jump from 1 to 18% B solution (0.1% formic acid and 93.75% acetonitrile) in 215 min, 18–28% in 30 min, and 28–95% in 20 min followed by a constant flow with 95% B for 35 min. The sample was introduced into a Thermo Lumos mass spectrometer and analyzed using a data dependent method, which collected a single MS1 spectrum at 120 K resolution setting (450–1500 m/z) with a normalized AGC target of 250% and maximum injection of 246ms on a seven second maximum cycle time during which MS2 spectra were collected in the ion trap. Monoisotopic peak determination was used, charge states of 2–4 were targeted and dynamic exclusion was used for two occurrences in 20 s (for 20 s). Data were analyzed using MaxQuant with the L (-CH3), M(-CD2H), and H(-13CD3) for amino- and lysine- labeling, no missed cleavage sites (to reduce variation from faint partially digested peptides), and advanced ratio estimation turned off.

### Cell toxicity assay

Astrocytes were infected with shRNA-expressing lentivirus for 72 h after plating. Cells were grown for a total of 7 days in serum-free medium supplemented with 5 ng/ml HBEGF in 6-well plate. Cells were then treated with 200 nM tau PFFs or an equivalent volume of DPBS (as a control) for 6 h, then washed extensively to remove remaining tau filaments. To further ensure that no Tau filaments were carried over to conditioned medium, cells were trypsinized and then re-seeded into a new 6-well plate, grown for an additional 48 h. At this time, the conditioned medium was collected, and a protease inhibitor cocktail was added to inhibit protein degradation. Conditioned medium was concentrated ~ 40 times by Amicon Ultra-15 Centrifugal Filter Units with 30 kDa cutoff filter (Millipore, UFC903024), and then added to iNeuron cultures (plated at 1.5 × 10^5^ cells per well in a poly-D-lysine-coated 24-well plate). The viability assay was performed 72 h later using the Live/Dead Kit (Thermo Fisher). Cells were imaged with a Nikon Eclipse TS100 fluorescence microscope equipped with a LWD 20 x/0.40 objective.

### Immunoprecipitation and immunoblotting

Immunoblotting was performed using a standard protocol. Proteins were separated by NuPAGE (4–12%) Bis-Tris gels (Thermo Fisher) and transferred onto nitrocellulose membranes (Bio-Rad). The target protein was detected by specific primary antibodies followed by secondary horseradish peroxidase (HRP)-conjugated antibodies (for less abundant proteins) (Sigma) or by fluorescence-labeled secondary antibodies (for abundant antigens) (Thermo Fisher). For immunoblotting with HRP-conjugated secondary antibodies, the signal was detected by the enhanced chemiluminescence method (ECL) using the Immobilon Western Chemiluminescent HRP substrate (Millipore) and recorded by a ChemiDoc™ MP Imaging System (Bio-Rad). The intensity of the detected protein bands was quantified by ImageLab v6.1 software (Bio-Rad). For immunoblotting with fluorescence-labeled secondary antibodies, membranes were scanned using a LI-COR Odyssey scanner. The intensity of the protein bands was quantified by the Odyssey software.

For cell surface binding and immunoprecipitation, one 10 cm petri dish of astrocytes were washed with pre-cold PBS two times to remove serum completely. We then prepared 200 nM tau PFF in serum-free pre-cold F-12 medium (3 ml for each 10 cm petri dish). We incubated cells with these proteins for 30 min at 4 ℃ with gentle shaking. After incubation, we removed the unbounded proteins by thoroughly washing 3 times. Cells were then lysed in 1 ml PBS-based lysis buffer with 0.5% NP40, a protease inhibitor cocktail, 1 mM DTT). After incubation on ice for 40 min, cell extracts were cleared by centrifugation at 15,000 g for 10 min at 4℃. Cleared supernatant was incubated with Tau46 antibody at 4 ℃ overnight to enrich interacting proteins. Protein A beads were added to the solution and further incubated at 4 ℃ with shaking for one hour. The beads were washed extensively with PBS containing 0.05% NP40. Bound proteins were eluted in 50 μl 1x SDS-PAGE Sample Buffer.

### Immunostaining with drug treatment

Primary astrocytes were seeded in 8 well Ibidi imaging chamber and treated with 200 nM tau PFF or OPN (20 μg/mL) for 6 h. Where indicated, cells were pre-treated with the following drugs: PF-562271 (1 μM), MK2206 (5 μM), PDTC (90 μM), PI103 (5 μM). The drugs were maintained during PFF treatment. Cells were washed with ice-cold PBS, fixed in 4% paraformaldehyde for 20 min at room temperature. Cells were then permeabilized in PBS containing 10% fetal bovine serum and 0.2% saponin for 10 min and then stained with antibodies against Calreticulin (1:100), GM130 (1:150), ERGIC53 (1:500) and VTI1B (1:100) at 4 °C overnight. Cells were rinsed 3 times with PBS before staining with fluorescence-labeled secondary antibody. Cells were imaged by a Nikon C1-Sora spinning disk confocal equipped with a 60x oil-immersion lens (NA 1.4). Images were processed by Imaris.(Oxford Instruments).

### Data and statistical analyses

RNAseq and mass spectrometry data were filtered by Excel. RNAseq experiments were all done with three independent biological repeats. Significant genes were then analyzed by STRING (https://string-db.org/) to identify protein interaction networks and biological processes enriched in selected gene cohorts. STRING-generated data were imported into Cytoscape to create the final protein interaction graphs. For quantitative mass spectrometry analyses of astrocyte secretome, since each sample requires a laborious fractionation step to maximize the coverage, only two independent biological repeats comprising of totally 60 fractions were analyzed. Promising candidates relevant to tau-induced astrogliosis were further confirmed by immunoblotting analysis in additional repeats. Quantification of fluorescence intensity or dot number was conducted using Image J. Immunoblotting data were quantified by Image Lab (Bio-Rad). The N values in figure legends indicate the number of independent biological replicates. Statistically analyses were performed using GraphPad Prism 9.0. For two-group comparison, unpaired Student’s t-test was used. For multi-group comparison, one-way ANOVA with Dunnett’s multiple comparison test was used.

### Electronic supplementary material

Below is the link to the electronic supplementary material.

Supplementary Material 1

Supplementary Material 2

Supplementary Material 3

Supplementary Material 4

Supplementary Material 5

Supplementary Material 6

Supplementary Material 7

Supplementary Material 8

## Data Availability

All data are available in the main text or the supplementary materials. The RNA sequencing data have been deposited to GEO under accession codes: GEO: GSE236868.
