# Supplementary material for: PI3K-AKT activation resculpts integrin signaling to drive filamentous tau-induced proinflammatory astrogliosis
Source: Cell Biosci. 2023 Sep 27;13:179. doi: 10.1186/s13578-023-01128-x (PMC10536728; doi:10.1186/s13578-023-01128-x)
Supplement: Supplementary file 6 — Supplementary Material 6 [file 13578_2023_1128_MOESM6_ESM.docx]

**Supplementary Figures**


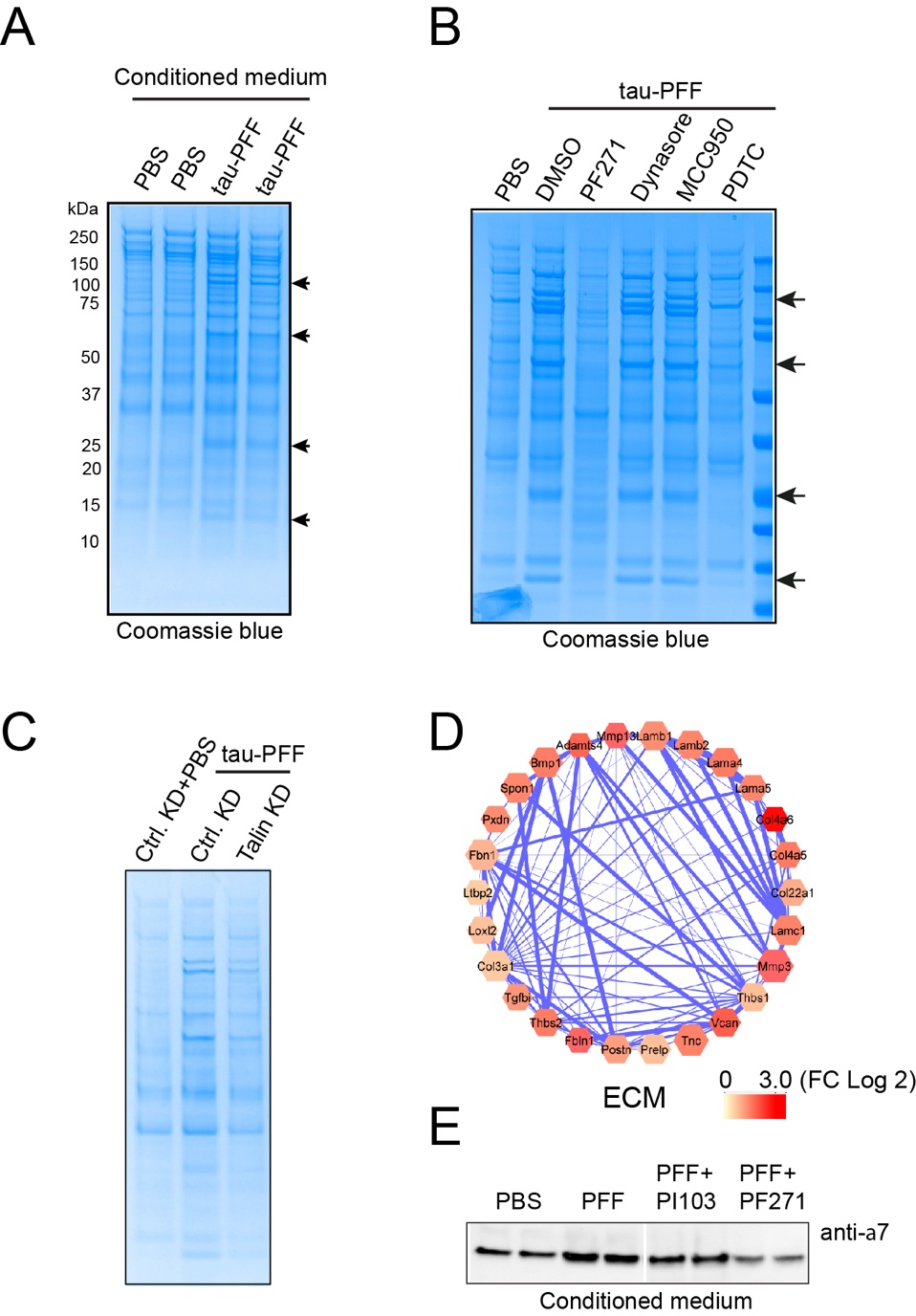


**Figure S1 Tau PFF induces protein secretion in immunopanning-purified astrocytes.**

**(A)** A Coomassie blue-stained gel showing the secreted proteins from immunopanning-purified PAs treated with tau PFF or PBS.

**(B)** As in (A), except that immunopanning-purified PAs were treated with PBS or with tau PFF together with the indicated agents. MCC950 is an inflammasome inhibitor and Dynasore is an endocytosis inhibitor. Note that these inhibitors have no effect on tau PFF-induced secretion.

The arrows in (A) and (B) indicate the significant bands only showed in condition medium from astrocytes treated with tau PFF.

**(C)** As in (A), except that control (Ctrl.) knockdown (KD) or Talin1 KD immunopanning-purified PAs were treated with tau PFF.

**(D)** The secretion of extracellular matrix proteins (ECM) was induced by tau PFF. Shown is an interaction network generated by Cytoscape. The color indicates fold change (FC).

**(E)** Immunoblotting confirms the secretion of the proteasome (as indicated by blotting the α7 subunit) from PAs. Note that α7 secretion was induced by tau PFF, which was reversed by the FAK inhibitor PF-562271 (PF271).

**
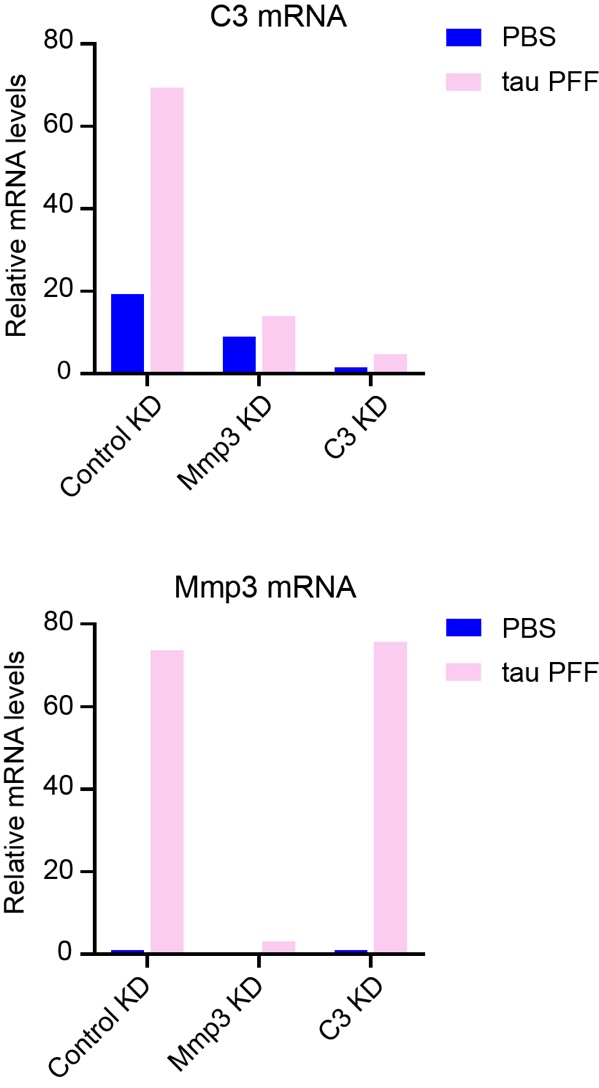
**

**Figure S2 qRT-PCR verification of Mmp3 and C3 knockdown in PAs.**

Tau PFF-induced expression of Mmp3 and C3 in control, Mmp3, or C3 knockdown (KD) cells. qRT-PCR was performed using mRNAs isolated from PAs treated with the indicated conditions.


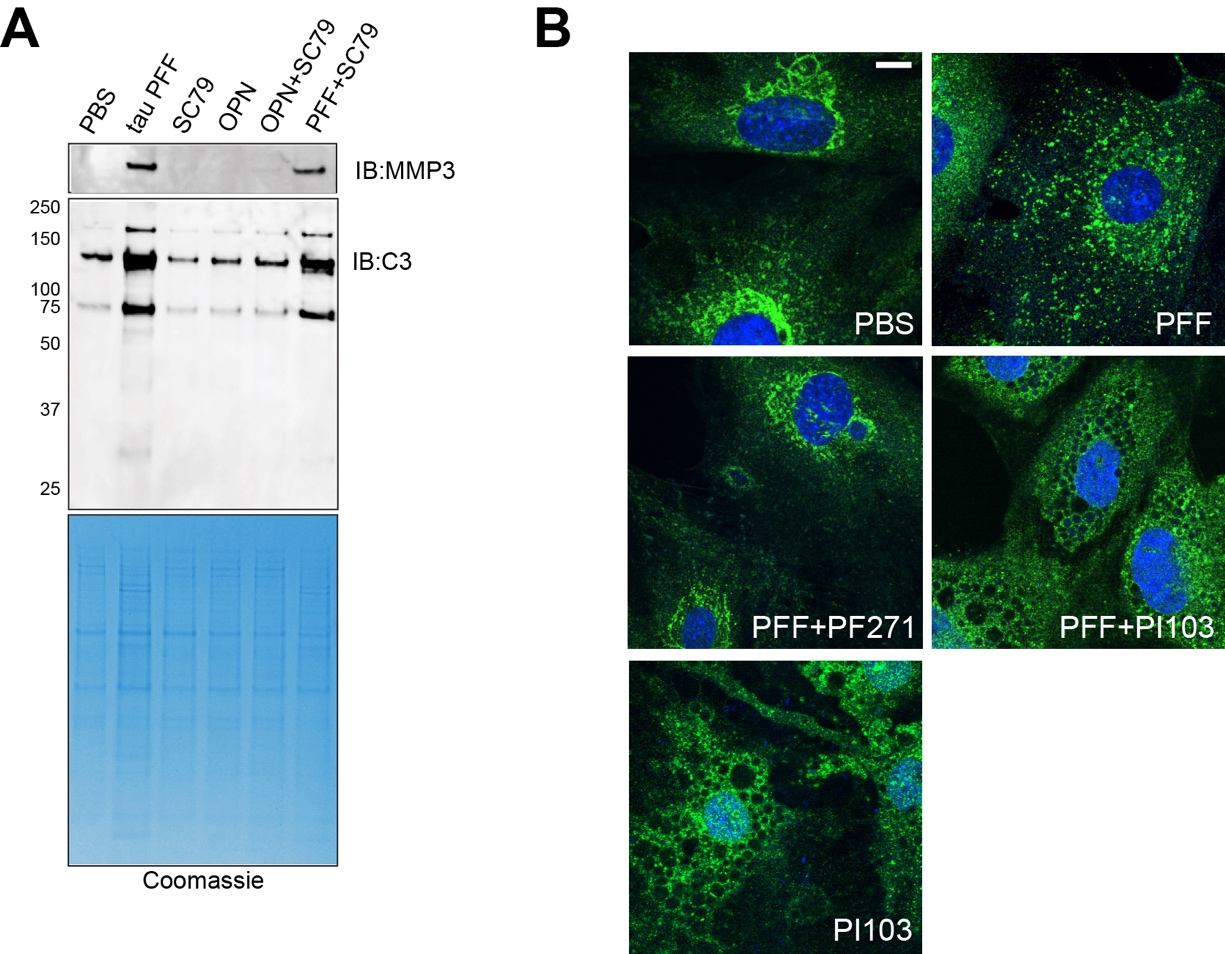


**Figure S3 tau PFF-induced fragmentation of the trans-Golgi network is inhibited by PF-562271 (PF271) but not PI103.**

(**A**) Coomassie blue staining (lower panel) and immunoblotting (IB) analysis (upper panels) of the conditioned medium from PAs treated as indicated.

(**B**) PAs treated with the indicated agents (6 h) were immunostained with anti-VTI1B antibodies (green) and Hoechst (blue) to label the trans-Golgi network and the nuclei, respectively. Scale bar, 10 µm. Note that PI103 treatment alone changes TGN, generating vacuole-like structures.


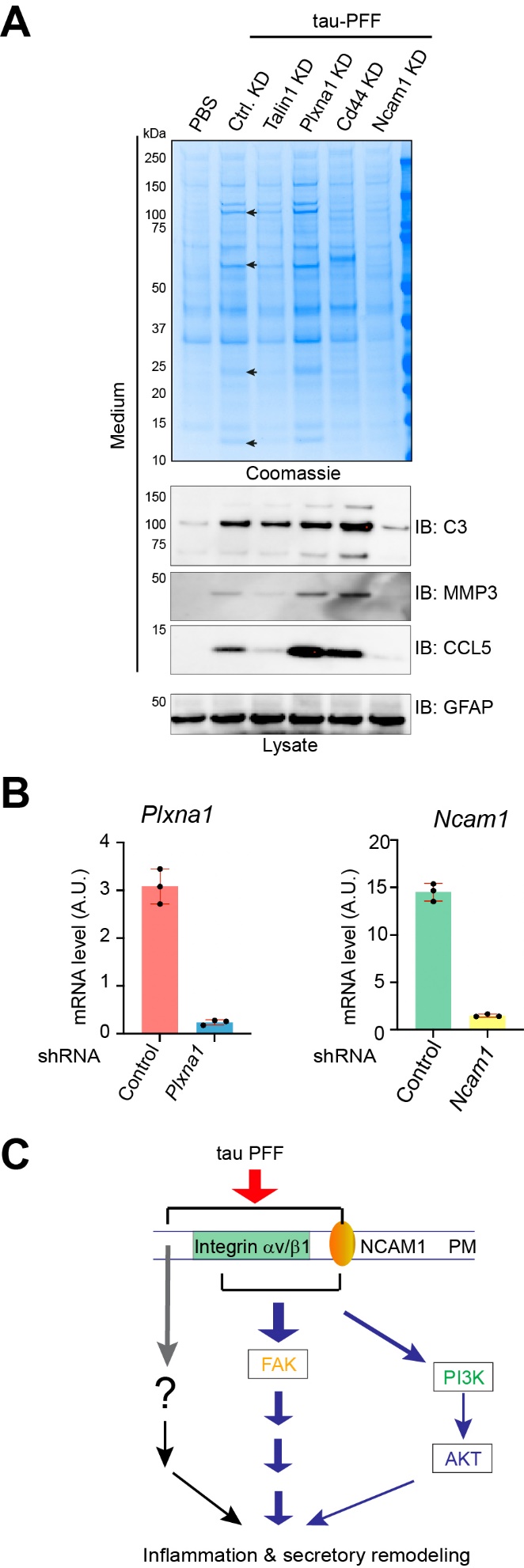


**Figure S4 NCAM1 is required for tau PFF-induced astrogliosis.**

**(A)** Tau PFF-induced protein secretion in PAs requires NCAM1. Conditioned medium of wild type PAs or cells with the indicated gene knockdown (KD) after exposure to tau-PFF. PBS-treated wild type astrocytes were used as a negative control. The arrows in (A) indicate significant protein bands only detected in tau PFF-treated condition medium.

**(B)** The gene knockdown efficiency of the indicated mRNAs was validated by qRT-PCR. n=3 biological repeats.

**(C)** A working model showing the mechanism underpinning tau PFF-induced pro-inflammatory astrogliosis and the remodeling of the secretory system. Tau PFF acts on both integrin αV/β1 and NCAM1 on the plasma membrane (PM), activating a signalosome that include multiple branches. In addition to FAK, tau PFF also activates PI3K-AKT and possibly other yet-to-be identified signaling process(es), which collectively convert astrocytes into a pro-inflammatory state.

**Supplementary tables**

**Table S1. The effect of tau PFF and OPN on gene expression in primary mouse astrocytes.**

The list contains significant genes (filtered by adjusted *p-value*<0.05) after tau PFF treatment for 6 h. Upregulated genes are shaded in yellow and downregulated genes in blue (FC > 2).

**Table S2. The effect of tau PFF and OPN on gene expression in primary mouse astrocytes.**

The list contains significant genes (filtered by adjusted *p-value*<0.05) after OPN treatment for 6 h. Upregulated genes are shaded in yellow and downregulated genes in blue (FC >2).

**Table S3. Genes differentially upregulated by tau PFF after 24 h treatment.**

The list contains genes whose upregulation is more significant in tau PFF-treated astrocytes than in OPN-treated ones.

**Table S4. The impact of various signaling inhibitors on tau PFF-induced gene expression changes.**

The list contains genes significantly affected by 6-h tau PFF treatment (adjusted *p-value* < 0.05) and also shows the impact of the indicated signaling inhibitors on the expression of these genes when combined with tau PFF treatment.

**Table S5. Tau PFF treatment upregulates protein secretion in primary astrocytes.**

The list contains proteins whose secretion is significantly induced by tau PFF treatment as determined by quantitative mass spectrometry analysis of conditioned medium.

**Table S6. The effect of tau PFF on the expression of genes in the complement system.**

Shown is a list of genes in the complement system with basal fpkm > 0.1.

**Table S7. A list of key reagents used in the study.**
